# Differences in peripheral and central metabolites and gut microbiome of laying hens with different feather-pecking phenotypes

**DOI:** 10.3389/fmicb.2023.1132866

**Published:** 2023-03-02

**Authors:** Chao Wang, Yaling Li, Haoliang Wang, Miao Li, Jinsheng Rong, Xindi Liao, Yinbao Wu, Yan Wang

**Affiliations:** ^1^Guangdong Provincial Laboratory of Lingnan Modern Agricultural Science and Technology, College of Animal Science, South China Agricultural University, Guangzhou, China; ^2^Guangdong Provincial Key Lab of Agro-Animal Genomics and Molecular Breeding, South China Agricultural University, Guangzhou, China; ^3^National Engineering Research Center for Breeding Swine Industry, South China Agricultural University, Guangzhou, China

**Keywords:** microbiota-gut-brain axis, laying hens, feather pecking, glutamatergic nerve system, metabolism, the commercial layer farm

## Abstract

**Background:**

Feather pecking (FP) is a maladaptive behavior in laying hens that is associated with numerous physiological traits, including those involving the central neurotransmitter system and the immune system, which have been identified in many species as being regulated by the gut microbiota *via* the “microbiota-gut-brain” (MGB) axis. Yet, it is unknown whether and how gut microbiota influences FP by regulating multiple central neurotransmission systems and immune system.

**Methods:**

This study was measured the prevalence of severe FP (SFP) in the commercial layer farm. The chicken flock with the highest frequency of SFP were selected for FP phenotype identification. Nontargeted metabolomics was performed to investigated the differences in the peripheral and central metabolites and 16S rDNA sequencing was performed to investigated the differences in gut microbiome of laying hens with different FP phenotypes. Correlation analysis was performed to determine the potential mechanism by which the disturbed gut microbiota may modulate host physiology and behavior.

**Results:**

The results showed that pullets (12 weeks of age) showed significantly higher SFP frequencies than chicks (6 weeks of age) and adults (22 weeks of age; *p* < 0.05). Compared to neutrals (N), peckers (P) exhibited the stress-induced immunosuppression with the increased plasma levels of corticosterone and norepinephrine, and the decreased plasma levels of IgA, IL-1, IL-6 and tumor necrosis factor α (*p* < 0.05). In the cecum, the relative abundances of *Bacteroides* and *Gemmiger* were higher in the P group, while *Roseburia*, *Ruminococcus2*, *Anaerostipes*, *Lachnospiracea_incertae_sedis* and *Methanobrevibacter* were more enriched in the N group. Moreover, increased plasma levels of L-tryptophan, beta-tyrosine and L-histidine were found in the P group (*p* < 0.05). Notably, in the P group, hippocampal levels of L-tryptophan, xanthurenic acid, L-histidine and histamine were improved and showed a positive association with L-glutamic acid levels. Plasma levels of L-tryptophan, beta-tyrosine and L-histidine were both positively correlated with *Bacteroides* abundance but negatively correlated with *Methanobrevibacter* abundance.

**Conclusion:**

Overall, these findings suggest that the development of FP may be affected by the gut microbiota, which regulates the central glutamatergic nerve system by altering the metabolism of tryptophan, histidine and tyrosine.

## 1. Introduction

Feather pecking (FP) is a maladaptive behavior with an identified prevalence of 80% in all laying hens housing systems (Gunnarsson, 1999). FP was divided into gentle feather peck (GFP) and severe feather pecking (SFP). SFP, a detrimental type of FP, can cause feather loss and skin damage, and in some cases, this can escalate to severe injuries and cannibalism, while GFP is suggested to be similar to social exploration without damage (Kops et al., 2013). What’s more, SFP can spread rapidly through learning among chickens, severely damaging animal welfare and causing economic losses (Rodenburg et al., 2013). Therefore, FP, especially SFP in laying hens is one of the most important unsolved behavioral issues in modern agriculture.

FP is multifactorial and has been linked to numerous behavioral characteristics, such as fearfulness, stress sensitivity and depression, but also to the central and peripheral physiological characteristics (Rodenburg et al., 2013). Deficiency or redundancy in the central serotonergic system can predispose birds to develop FP, while birds with high FP tendency generally have low rates of central serotonin (5-HT) and dopamine (DA) turnover at a young age but high turnover in the brain at an adult age (de Haas and van der Eijk, 2018). The brain transcriptomes of laying hens divergently selected for FP reveal the potential role of GABAergic and glutamatergic neurotransmitter systems in the development of FP (Falker-Gieske et al., 2020). Birds selected for high FP (HFP) and low FP (LFP) differ in innate and adapted immune characteristics (van der Eijk et al., 2019a,b). These studies above suggest that the occurrence of FP may be related to alterations in multiple central neurotransmission systems and immune system, however, there is a lack of clear evidence for the cause of these alterations.

The gastrointestinal tract is a complex ecosystem containing a large number of resident microorganisms that have been found to play an important role in the maintenance of host behavior *via* the “microbiota-gut-brain” (MGB) axis (Zheng et al., 2019). Recent evidence suggests that alterations in gut microbiota composition, *via*, for example, anti-, pre-or probiotic treatment, affect anxiety, stress sensitivity and fearfulness (Desbonnet et al., 2010; Ait-Belgnaoui et al., 2014). Moreover, the regulatory effects of the gut microbiota on serotonergic, dopaminergic, GABAergic and glutamatergic neurotransmitter systems in the central nervous system (CNS) and immune system have also been identified by many studies (Miller et al., 2011; Zheng et al., 2019; Hasebe et al., 2021). Notably, a growing number of studies are focusing on the potential role of the regulation of the gut microbiota in FP development. Laying hens that are divergently selected for FP (HFP and LFP) show significant differences in gut microbiota composition (Birkl et al., 2018; van der Eijk et al., 2019c). Early-life transplantation of microbiota from HFP birds influences the behavioral and physiological characteristics that are related to FP (van der Eijk et al., 2020). Collectively, these findings highlight the novel possibility that disturbances of gut microbiota or the MGB axis may contribute to the onset of FP in laying hens. Yet, it is unknown whether and how gut microbiota influences FP by multiple central neurotransmission systems and immune system. Metabolism is an important pathway of two-way communication between gut microorganisms and the brain (Zheng et al., 2019), however, there is a lack of systematic studies on the metabolism in laying hens with different FP phenotypes (including pecker, victim and neutral).

Outcomes from numerous research methods, including improving the house climate (light intensity, temperature, humidity and sound) and foraging condition (rearing intensity, feed shape and nonstarch polysaccharide concentration), indicate that the appropriate rearing environment plays a key role in alleviating the development of FP in laying hens (Lambton et al., 2010; Gilani et al., 2013). The physically, nutritionally, sensorially and socially restricted environment in which the majority of commercial laying hens hatch and live can be a powerful social and environmental chronic stressor that could induce a high occurrence rate of SFP (Maes et al., 2009). Therefore, the aim of this research was to investigate the prevalence of SFP in the commercial layer farm, as well as the metabolic characteristics and the gut microbial characteristics of laying hens with different FP phenotypes, and to reveal the potential correlation between gut microbiota and FP.

To address this issue, the frequency of SFP in the three stages of laying hens, which include chick, pellet and adult was investigated in a commercial layer farm. The chicken flock with the highest frequency of SFP were selected for FP phenotype identification and laboratory analysis. 16S ribosomal RNA (16S rRNA) gene sequencing analysis was performed to reveal the difference in the gut microbial communities of laying hens with different FP phenotypes. We conducted nontarget metabolomic analysis of the plasma and hippocampus to investigate metabolic changes in the peripheral and central systems. Finally, correlation analysis was performed to determine the potential mechanism by which the disturbed gut microbiota may modulate host physiology and behavior.

## 2. Materials and methods

### 2.1. Animals and housing conditions

**Figure 1:**
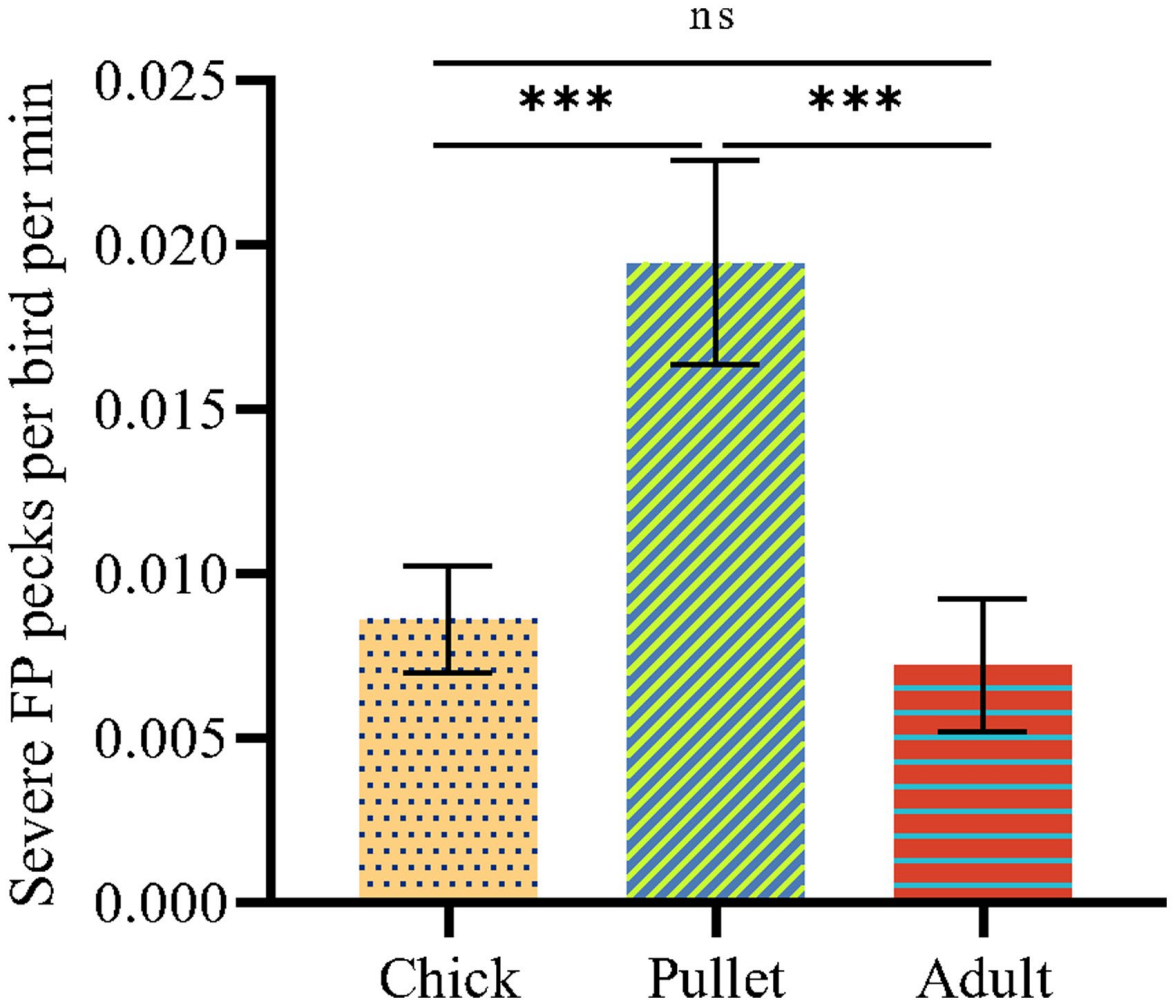
The SFP frequencies of birds at different stage of laying hens. The statistically significant differences between different groups are indicated as asterisks (*p < 0.05, **p < 0.01, and ***p < 0.001).

This research was conducted in a commercial layer farm (Guangdong Lvyang Agricultural Co., LTD, Guangdong Province, China). First, preliminary FP observation was conducted for three consecutive days to select the chicken flock with the highest frequency of SFP among three different stages of chicken flocks (chick, pellet and adult stages of chicken) of beak-trimming Hyline gray laying hens. Specifically, 6 cages were randomly selected from laying hens at 6 weeks of age (40 chicks per cage), 12 weeks of age (25 pullets per cage) and 22 weeks of age (10 adults per cage), and pullet flocks at 12 weeks of age showed more SFP (Figure 1). Therefore, a total of 200 12-weeks-old beak-trimmed birds (8 cages, 25 birds per cage) were then selected, individually identified using numbered silicone backpacks (6 × 6 × 0.5 cm; Birkl et al., 2017) and transferred to the top cage without disturbing the flock. Birds were kept in battery layer cages (120 l*60 W*45 H cm for chicks, 100 l*50*45 H cm for pullets and 120 l*60 W*45 H cm for adults) and reared under conventional management conditions on a commercial farm. One camera (HIKVISION DS-2CD2T55(D)-I3, Hangzhou, China) was installed 1 meter above each cage to enable a full view of the cage. The cages were arranged at intervals, so it was certain that there was no visual contact between the different cages. At 6 weeks of age, the light was on for 9 h, from 8:00 until 17:00. At 12 weeks of age, the light was provided from 6:00 to 22:00, and this stayed the same throughout the laying period. Birds had *ad libitum* access to well water and commercial layer feed. The animals used in this study were treated in accordance with the approval of the Scientific Ethics Committee of South China Agricultural University under approved permit number SYXK2014-0136.

### 2.2. Behavioral observations

For the preliminary FP observation, each cage was video recorded for 10 min (1 × in the morning between 8 and 11 am, 1 × in the after between 3 and 5 pm) on 3 consecutive days. FP was divided into GFP (subdivided into exploratory FP and stereotyped FP) and SFP as adapted from (Birkl et al., 2017). SFP was defined as follows: “A bird grips and pulls or tears vigorously at a feather of another bird with her beak, causing the feather to lift up, break or be pulled out. The recipient reacts to the peck by vocalizing, moving away or turning toward the pecking bird.” The number of SFP events was observed using the video and recoded at the cage level. After individual identification and transfer, birds were provided 2 weeks before the experiment started. Backpacks were fastened around the wings *via* two elastic straps secured to the backpacks with metal eyelets (Mindus et al., 2021). At 14 weeks of age, FP behavior was observed at an individual level. SFP was observed from video recordings, and each observation lasted 20 min and was performed once in the morning (8:30–11:30) and once in the afternoon (14:00–17:00). The number of SFP events, either given or received, was summed over 4 consecutive days, thus including one morning and one afternoon observation with a total observation period of 40 min, and was used to identify FP phenotypes (Daigle et al., 2015). When a bird gave more than one and received zero or one severe FP, it was defined as a pecker (P). When a bird gave and received zero or one severe feather peck, it was defined as neutral (N). We did not include victims or feather pecker-victims in this study. All-occurrence sampling was used to record initiators and recipients of SFP interactions. An occurrence was defined as a sequence of uninterrupted behavior lasting more than 4 s aimed at the same bird (Birkl et al., 2017). All behavioral observations were performed by a trained, blinded observer.

### 2.3. Sample collection

After FP observation, one pecker and one natural were chosen from each cage, for a total of 16 hens. Blood samples were collected from the wing vein using EDTA-coated vacutainer tubes. Blood samples were stored on ice (maximum of 4 h) until centrifugation (4°C, 2,500 rpm, 15 min) for plasma separation. Plasma was aliquoted into 1.5 ml microtubes and stored at-80°C until further determination of plasma stress and immune indices. After blood sampling, the birds were euthanized by cervical dislocation to obtain the contents of the duodenum, ileum and caeca. Gut contents were stored in 2 ml cryovials at-80°C until further analysis. The hippocampus, considered the memory and learning area in both mammals and birds, was quickly sampled on ice and stored in liquid nitrogen (Colombo and Broadbent, 2000).

### 2.4. Measurement of plasma stress and immune indices

The plasma levels of interleukin-1 (IL-1), interleukin-6 (IL-6), immunoglobulin A (IgA), immunoglobulin G (IgG), tumor necrosis factor α (TNF-α), and corticosterone (CORT) (Item No#: YJ059829, YJ042757, YJ002792, YJ042771, YJ002790, YJ059881, Shanghai Enzyme Linked Biotechnology Co., Ltd., Shanghai, China) were measured using a commercially available ELISA kit following the manufacturer’s protocol. The optical density of each sample was read at 450 nm using Nessler’s reagent spectrophotometry (Shanghai Ao Yi Technology Co., Ltd., Shanghai).

### 2.5. DNA extraction and 16S rRNA gene sequencing

The total bacterial DNA of each sample of gut content was extracted using the QIAamp PowerFecal Pro DNA Kit (Qiagen, Hilden, Germany) following the manufacturer’s instructions. DNA quality was examined by electrophoresis on a 1% agarose gel, and the final DNA concentration of each sample was determined using an ultrafine, ultraviolet spectrophotometer (Shanghai Ao Yi Technology Co. Ltd., Shanghai). DNA samples were stored at-80°C until further analysis.

The 16S rRNA gene amplicons were used to determine diversity and compare the structures of bacterial communities among samples to reveal taxonomic composition. Next-generation sequencing library preparations and Illumina NoveSeq sequencing were conducted at Novogene Co., Ltd., Tianjin, China. The V3-V4 hypervariable regions of the 16S rRNA genes were amplified with forward primers containing the sequence 5’-CCTAYGGGRBGCASCAG-3′ and reverse primers containing the sequence 5’-GGACTACNNGGGTATCTAAT-3′. Furthermore, indexed adapters were added to both ends of the 16S rDNA amplicons to generate indexed libraries ready for downstream NGS sequencing on the Illumina NovaSeq platform. The quality of each DNA library was validated with an Agilent 2,100 Bioanalyzer (Agilent Technologies, Palo Alto, CA, United States), and the concentration was measured by a Qubit 2.0 Fluorometer (Thermo Fisher Scientific, Carlsbad, CA, United States). DNA libraries were multiplexed and loaded on an Illumina NovaSeq6000 instrument according to the manufacturer’s instructions (Illumina, San Diego, CA, United States). Sequencing was performed using the 2 × 300 bp paired-end configuration. Image analysis and base calling were performed by NovaSeq Control Software embedded in the NovaSeq instrument.

### 2.6. Nontargeted metabolomics

Thawed plasma samples at 4°C were vortexed for 1 min after thawing and mixed evenly as described in previous research (Zelena et al., 2009) with some modifications. For metabolite extraction, cold methanol (stored at-20°C) was added at a ratio of 400 μl per 100 μl of plasma. The samples were then vortexed for 1 min and centrifuged for 10 min at 4°C and 13,000 rpm. The samples were kept on ice between the steps. The supernatant was transferred to a new 2 ml centrifuge tube and dissolved in 150 μl of 2-chloro-l-phenylalanine (4 ppm) solution prepared with 80% methanol water (stored at 4°C). The supernatant was filtered with a 0.22 μm membrane and inserted into HPLC vials for analysis. To extract hippocampal metabolites, samples weighing 60 mg were ground at 50 Hz for 1 min in 1 ml of cold tissue extract (75% 9:1 methanol: chloroform, 25% H_2_O) with 3 steel balls (Want et al., 2013). After room temperature ultrasound for 30 min and ice bath for 30 min, the samples were centrifuged at 12000 rpm and 4°C for the supernatant. The supernatant was then redissolved with 200 μl of 50% acetonitrile solution prepared with 2-amino-3-(2-chlorophenyl)-propionic acid (4 ppm; stored at 4°C) and filtered through a 0.22 μm membrane for Liquid chromatography-mass spectrometry (LC–MS) detection.

All samples were analyzed by LC–MS. Liquid chromatography (LC) analysis was performed on a Vanquish UHPLC System (Thermo Fisher Scientific, United States). Chromatography was carried out with an ACQUITY UPLC ® HSS T3 (150 × 2.1 mm, 1.8 μm; Waters, Milford, MA, United States). The column temperature was maintained at 40°C. The flow rate and injection volume were set at 0.25 ml/min and 2 μl, respectively. For LC-ESI (+)-MS analysis, the mobile phases consisted of (C) 0.1% formic acid in acetonitrile (v/v) and (D) 0.1% formic acid in water (v/v). Separation was conducted under the following gradient: 0 ~ 1 min, 2% C; 1 ~ 9 min, 2% ~ 50% C; 9 ~ 12 min, 50% ~ 98% C; 12 ~ 13.5 min, 98% C; 13.5 ~ 14 min, 98% ~ 2% C; 14 ~ 20 min, 2% C. For LC-ESI (−)-MS analysis, the analytes were carried out with (A) acetonitrile and (B) ammonium formate (5 mM). Separation was conducted under the following gradient: 0 ~ 1 min, 2% A; 1 ~ 9 min, 2% ~ 50% A; 9 ~ 12 min, 50% ~ 98% A; 12 ~ 13.5 min, 98% A; 13.5 ~ 14 min, 98% ~ 2% A; 14 ~ 17 min, 2% A. Mass spectrometric (MS) detection of metabolites was performed on a Q Exactive (Thermo Fisher Scientific, United States) with an ESI ion source. Simultaneous MS1 and MS/MS (Full MS-ddMS2 mode, data-dependent MS/MS) acquisition was used. The parameters were as follows: sheath gas pressure, 30 arb; aux gas flow, 10 arb; spray voltage, 3.50 kV and-2.50 kV for ESI(+) and ESI(−), respectively; capillary temperature, 325°C; MS1 range, m/z 81–1,000; MS1 resolving power, 70,000 FWHM; number of data-dependent scans per cycle, 10; MS/MS resolving power, 17,500 FWHM; normalized collision energy, 30%; dynamic exclusion time, automatic.

### 2.7. Statistical analysis

#### 2.7.1. Behavior observation data analysis

The SFP frequencies were determined per individual cage per min and averaged at the cage level. IBM SPSS Statistic 26 software (IBM Corp., Armonk, NY) was used to compare the SFP frequencies by one-way analysis of variance (ANOVA). Multiple comparisons were conducted using the Duncan method. The data are presented as the means ± standard errors (SE). Significant differences were reported as those with *p* < 0.05.

#### 2.7.2. Plasma stress and immune data analysis

The plasma stress and immune data were analyzed with independent sample T tests using IBM SPSS Statistic 26 software (IBM Corp., Armonk, NY). The data are presented as the means ± SE. Significant differences were reported as those with *p* < 0.05.

#### 2.7.3. 16S rRNA gene sequencing data analysis

The 16S rRNA gene sequencing data were analyzed using the QIIME data analysis package (V1.9.1^1^) in R software (version 4.0.3) and R studio (version 1.3.1093). The forward and reverse reads were joined to form joined sequences. After removing barcode and primer sequences, the reads of each sample were spliced using FLASH V 1.2.7.^2^ Quality filtering of the joined sequences was performed. Sequences that did not fulfill the following standards were discarded: sequence length < 200 bp, no ambiguous bases, and mean quality score ≥ 15. The remaining sequences were compared with the reference database (RDP Gold database) using the UCHIME algorithm^3^ to detect chimeras. Sequences with chimeric sequences were removed from further analysis. Filtered sequences were grouped into operational taxonomic units (OTUs) using the clustering program VSEARCH (V2.15.1^4^) against the Silva 119 database at 97% sequence identity. The Ribosomal Database Program (RDP) classifier was used to assign taxonomic categories to all OTUs at a confidence threshold of 0.8. The α and β diversity analyzes were conducted using USEARCH (V10.0.240^5^).

Alpha diversity (α-diversity) indices, including the Shannon index for diversity^6^ and the Chao1 index for richness,^7^ were calculated by QIIME (1.9.1) from rarefied samples. Difference analyzes of the alpha diversity index, parametric tests and nonparametric tests were conducted. Because there were only two groups, the T test was used for the difference analysis of the alpha diversity index. Beta diversities were calculated using unweighted UniFrac distances. The unweighted pair group method with arithmetic mean was used to generate dendrograms from the beta diversity distance matrix. The principal coordinate analysis (PCoA) and the significance analysis of microbial structure were performed using the OmicStudio tools at https://www.omicstudio.cn/tool. The PCoA analysis was performed based on the unweighted unifrac distance and the *p* value was calculated using the analysis of similarity (ANOSIM). The key bacterial taxa responsible for discrimination between the two groups were identified using linear discriminant analysis (LDA) effective size (LEfSe).^8^ The threshold of the logarithmic LDA score was 3.5.

#### 2.7.4. Nontargeted metabolomic data analysis

The raw data were first converted to mzXML format by MSConvert in the ProteoWizard software package (v3.0.8789) and processed using XCMS for feature detection, retention time correction and alignment. The metabolites were identified by accuracy mass (< 30 ppm) and MS/MS data, which were matched with HMDB,^9^ massbank,^10^ LipidMaps,^11^ mzclound^12^ and KEGG.^13^ The robust LOESS signal correction (QC-RLSC) was applied for data normalization to correct for any systematic bias. After normalization, only ion peaks with relative standard deviations (RSDs) less than 30% in QC were kept to ensure proper metabolite identification.

Ropls software was used for all multivariate data analyzes and modeling. Data were mean-centered using scaling. Models were generated on principal component analysis (PCA), orthogonal partial least-square discriminant analysis (PLS-DA) and partial least-square discriminant analysis (OPLS-DA). The metabolic profiles could be visualized as a score plot, where each point represents a sample. The corresponding loading plot and S-plot were generated to provide information on the metabolites that influence the clustering of the samples. All the models evaluated were tested for overfitting with permutation tests. The descriptive performance of the models was determined by R2X (cumulative; perfect model: R2X (*cum*) = 1) and R2Y (cumulative; perfect model: R2Y (*cum*) = 1) values, while their prediction performance was measured by Q2 (cumulative; perfect model: Q2 (*cum*) = 1) and a permutation test. The permuted model should not be able to predict classes: R2 and Q2 values at the Y-axis intercept must be lower than those of Q2 and the R2 of the nonpermuted model. OPLS-DA allowed the determination of discriminating metabolites using the variable importance on projection (VIP). The *p* value, variable importance projection (VIP) produced by OPLS-DA and fold change (FC) were applied to discover the contributable variables for classification. Finally, results with a *p* value < 0.05 and VIP values > 1 were considered to be statistically significant metabolites. Differential metabolites were subjected to pathway analysis by MetaboAnalyst,^14^ which combines results from powerful pathway enrichment analysis with pathway topology analysis. The metabolites identified in the metabolomics analysis were then subjected to KEGG pathway analysis for biological interpretation of higher-level systemic functions. The metabolites and corresponding pathways were visualized using the KEGG Mapper tool.

## 3. Results and discussion

### 3.1. Twelve-week-old birds showed more serious SFP

The SFP frequencies of birds at different stages are shown in Figure 1. Pullets showed significantly higher SFP frequencies than chicks and adults (*p* < 0.05). Compared to adults, pullets were kept at a higher rearing density. A previous study identified that the high rearing density of laying hens results in FP at the pullet stage and not the adult stage in hens (30 weeks of age) on commercial farms (Bestman et al., 2009). In this study, in addition to evaluating rearing density, continuous inspection and isolation of victims during commercial farming were investigated and may have been a potential factor contributing to the decrease in SFP at the adult stage of laying hens. The pullet stage is the stage of fastest weight gain, with higher stress vulnerability and more serious SFP than in chicks (Rodenburg et al., 2004). Therefore, pullets were selected for further investigation in this study.

### 3.2. Stress-induced immunosuppression in peckers

**Figure 2:**
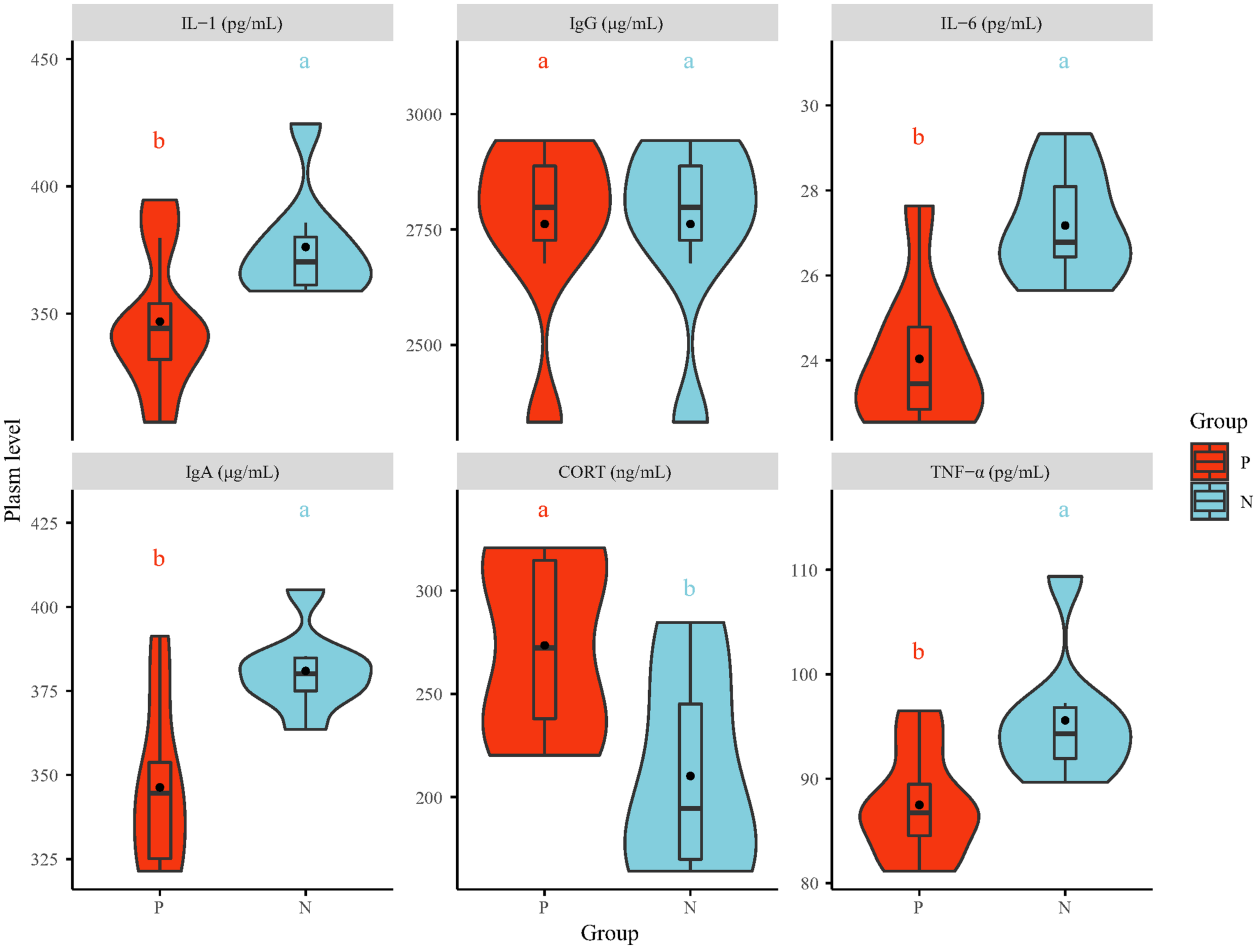
The plasma level of stress (CORT) and immune indices (IgA, IgG, IL-1, IL-6 and TNF-α). IgA = Immunoglobulin A, IgG = Immunoglobulin G, IL-1 = interleukin-1, IL-6 = interleukin-6, TNF-α = tumor necrosis factor-α, CORT = corticosterone. The values in the violin plot are the means ± SE (*n* = 8). The statistically significant differences between the P and the N group are indicated as different letter (a, b; *p* < 0.05). P = feather pecker, N = neutra.

After 4 consecutive days of behavioral observation, one pecker and one natural were chosen from each cage, resulting in a total of 16 hens, for further investigation. The plasma levels of stress and immune indices were measured by ELISA (Figure 2). The plasma levels of IgA, IL-1, IL-6 and TNF-α in the P group were significantly lower than those in the N group (*p* < 0.05), while the IgG level showed no significant difference. Plasma CORT and Norepinephrine (NE) (Supplementary Figure S6) levels, which are measured in bird stress indices, were increased in the P group (*p* < 0.05). The increased stress hormone levels measured in the P group suggest that the FP birds were in a state of stress, and stress hormone signaling has been identified as the final common pathway involved in regulating the pathophysiological status and behavior of animals (Dallman et al., 1994). Various unpredicted extreme or mild chronic stresses, such as noise, mixing chicken breeds, strengthening or weakening light intensity and chicken transfer, have been found to contribute to the development of FP in chickens (Maes et al., 2009). The outcomes from numerous experiments indicate that unpredicted stress is associated with many detrimental behaviors, including depression-like and anxiety-like behavior, *via* the immune system in both humans and animals (Ait-Belgnaoui et al., 2014). In this research, the suppressed immune system, which was associated with decreased levels of IgA and proinflammatory cytokines (IL-1, IL-6 and TNF-α), may have been the result of central anti-inflammatory cytokines (IL-10) expression caused by long-term stress (Mormède et al., 2003). Moreover, elevated stress hormone levels can lead to disrupted gut barrier function and altered commensal bacteria (Maes et al., 2013).

### 3.3. Changes in the intestinal microbiota community in peckers

**Figure 3:**
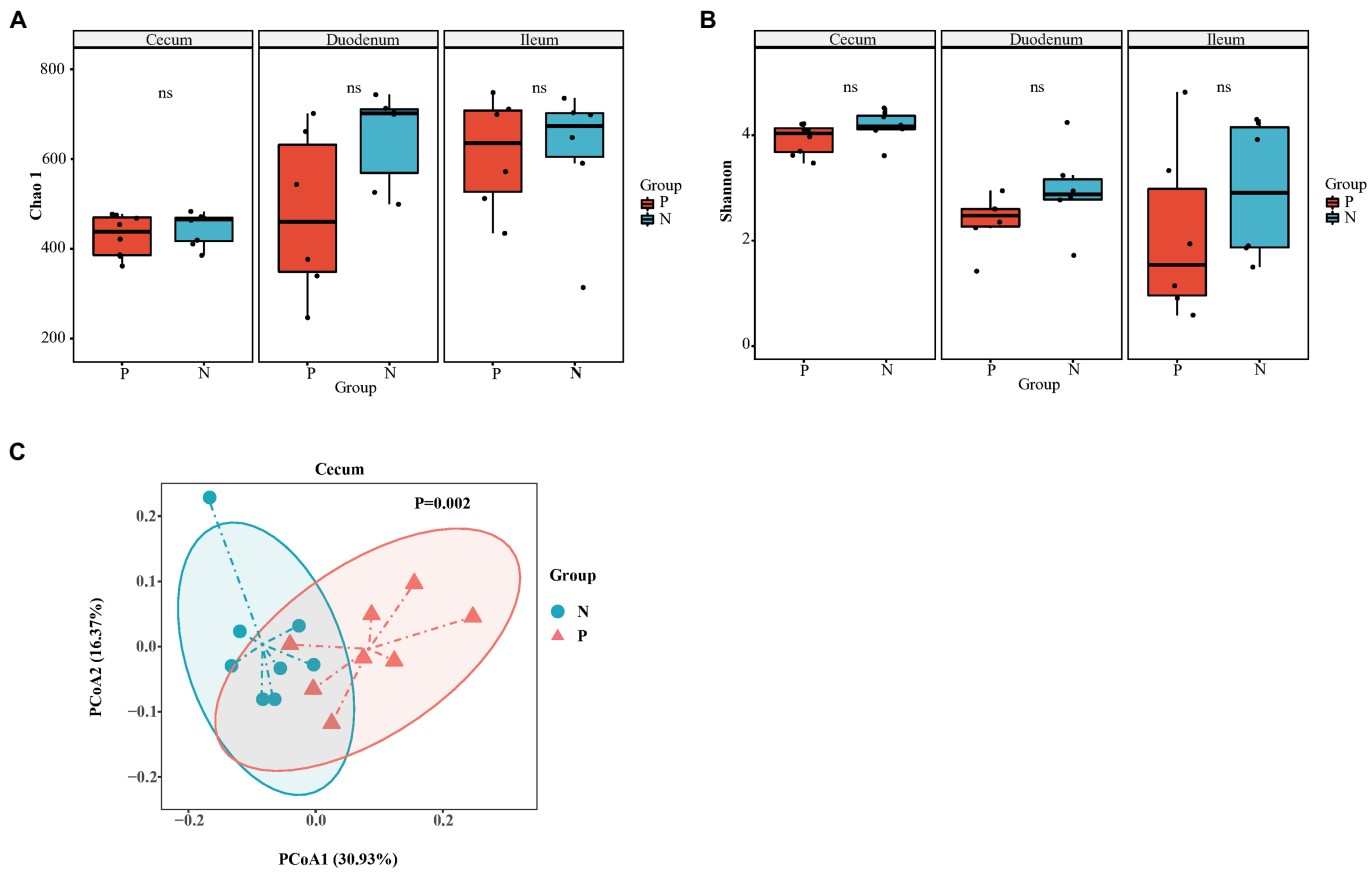
The diversity of gut microbiota of laying hen with different FP phenotype. **(A)** Chao 1 index of cecum (*n* = 8), duodenum (*n* = 6) and ileum (*n* = 6); **(B)** Shannon index of cecum, duodenum and ileum; **(C)** The principal co-ordinates (PCoA) analysis of cecum based on the unweighted unifrac distance. P = feather pecker, N = neutral.

**Figure 4:**
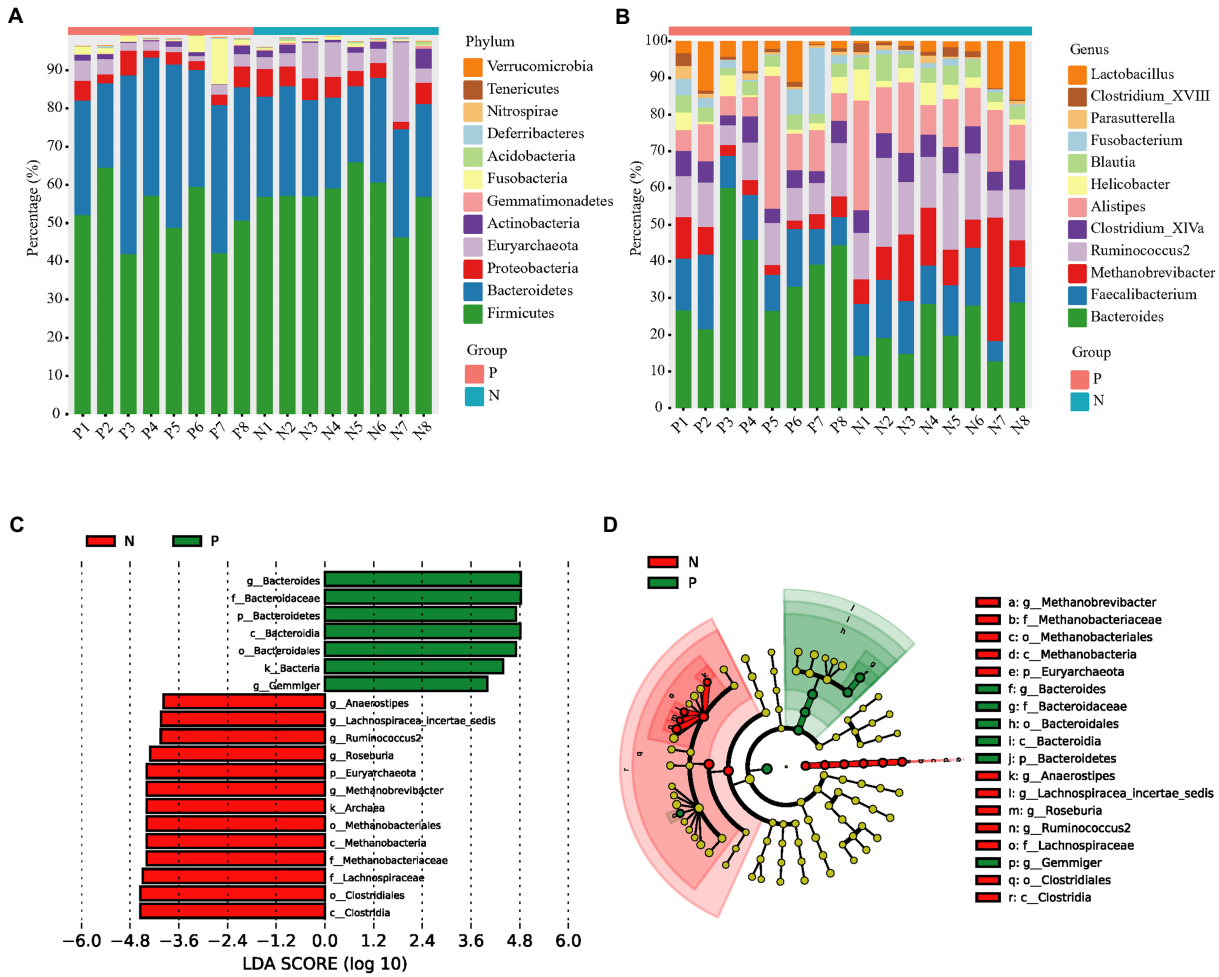
The relative abundance of cecal bacterial composition at the phylum and genus level and LefSe analysis of cecal microbiota. **(A)** The Relative abundance of cecal bacterial composition at phylum level; **(B)** The Relative abundance of cecal bacterial composition at genus level; **(C)** and **(D)** LEfSe analysis of cecal microbiota. The threshold of the logarithmic LDA score was 3.5. P = feather pecker, N = neutral.

In total, we obtained 967,536 high-quality reads across all cecal samples, and these reads were clustered into 509 OTUs at 97% sequence similarity. In the duodenum and ileum, 809,736 and 785,292 high-quality sequences and 788 and 817 OTUs were obtained, respectively. Most rarefaction curves tended to approach the saturation plateau, suggesting that the sequencing depth was sufficient to cover the whole bacterial diversity (Supplementary Figures S1A–C). Alpha diversity (α-diversity) showed that both the richness index (Chao 1) and diversity index (Shannon) of the duodenum, ileum and cecum did not differ between the P group and the N group (Figures 3A,B). To determine whether the microbial composition of birds with FP was substantially different from that of the N group, we carried out β-diversity analysis. Based on the unweighted UniFrac distance, PCoA revealed a significant difference in the cecal microbiota community (*p* < 0.05) but no significant difference in the duodenum and ileum (*p* > 0.05; Figure 3C; Supplementary Figures S2A,B). Therefore, we focused on the characteristics of the cecal microbial community in the rest of this study. At the phylum level, the top five phyla identified were *Firmicutes*, *Bacteroidetes*, *Proteobacteria*, *Euryarchaeota* and *Fusobacteria* in the cecum of laying hens (Figure 4A). Among these phyla, *Firmicutes* and *Bacteroidetes* were dominant. At the genus level, the majority of 16S rRNA amplicons belonged to *Bacteroides*, *Faecalibacterium*, *Methanobrevibacter*, *Ruminococcus2*, *Alistipes* and *Lactobacillus* (Figure 4B). To further identify the cecal microbiota responsible for discriminating peckers from neutrals, we carried out LEfSe. This analysis identified 7 differential microorganisms responsible for the discrimination between the two groups at the genus level (Figures 4C,D). The relative abundances of *Bacteroides* and *Gemmiger* were higher in the P group, while *Roseburia*, *Ruminococcus2*, *Anaerostipes*, *Lachnospiracea_incertae_sedis* and *Methanobrevibacter* were more enriched in the N group (*p* < 0.05). These data imply that the structure of the cecal microbiota was disordered in peckers.

The gut microbiota, known as the second brain, has been found to exert its effect on the brain and behavior by regulating catabolism or the nervous system based on the “gut-brain axis” (Zheng et al., 2019). To date, an increasing number of investigations have focused on whether gut microbiota affect feather pecking in laying hens. Early-life microbiota transplantation showed a long-term influence on depression-like and anxiety-like behavior related to FP in laying hens (van der Eijk et al., 2020). The gut microbiota analysis of LFP and HFP birds revealed significantly different diversity and composition, including increased abundanc**e** of *Lactobacillus* and decreased abundance of *Clostridiales* in HFP birds (van der Eijk et al., 2019c). Moreover, ingestion of *Lactobacillus rhamnosus* retards chronic stress-induced FP in chickens, suggesting the important role of gut microorganisms in relieving FP in birds (Mindus et al., 2021). The features of the gut microbiota found in this research showed a different pattern from those observed in previous studies, which could have been the result of multiple factors, including genetics, nutrition and stress (Maes et al., 2013; Tremblay et al., 2021). *Bacteroides*, *Ruminococcus2* and *Methanobrevibacter*, the main enriched microorganisms, were found to have a higher or lower relative abundance in peckers, which has a close association with depression-like and anxiety-like behavior (Chen et al., 2021; Zhang et al., 2022). Knowledge regarding the underlying associations between feather pecking and these differential microbes are vague and require further metabolomics analysis.

### 3.4. Changes in the metabolic profile in peripheral and central organisms in peckers

**Figure 5:**
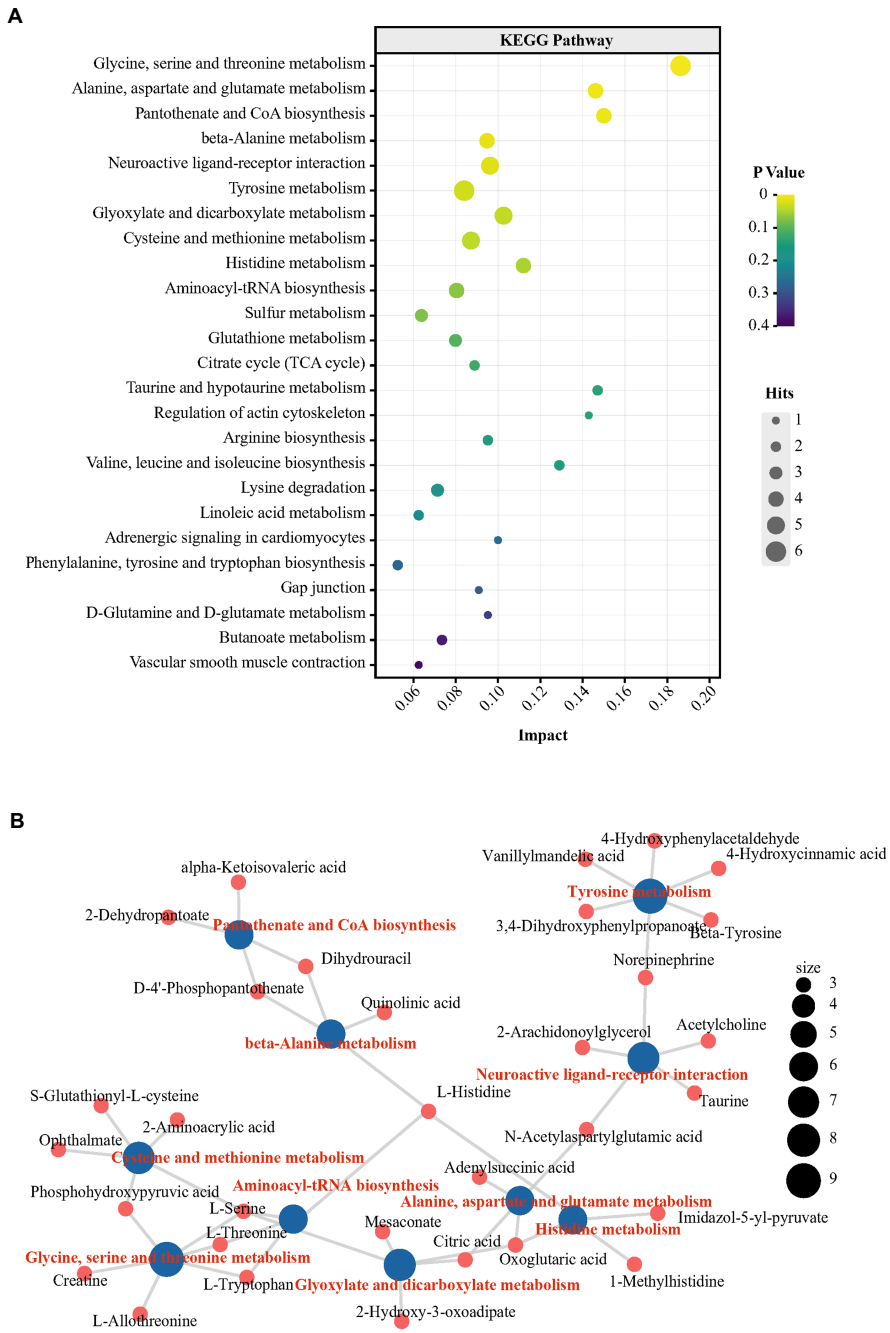
Changes in the metabolic profile in plasma obtained from FP birds. **(A)** Bubble chart using the top 25 KEGG pathways enriched by the differential metabolites in plasma; **(B)** Network plot using the top 10 KEGG pathways enriched from the differential metabolites in plasma.

We further performed nontargeted metabolomics to determine whether the metabolic state reflected in the plasma and hippocampus were paralleled by an altered gut microbiota. To further explore the metabolic distinctions between the P and N groups, the multivariate statistical analysis was performed (Supplementary Figures S3–S5). Furthermore, a total of 94 metabolites were changed significantly in the P groups. The P group had 89 metabolites with higher levels and 5 metabolites with lower levels than in the N group (*p* < 0.05; Supplementary Figure S6; Supplementary Table S1). Tryptophan, as the precursor of the central major inhibitory neurotransmitter 5-HT, can pass through the blood brain barrier (BBB) and affect brain function and behavior (Hasebe et al., 2021). Tryptophan-5-HT deficiency has been identified to be involved in the development of many maladaptive behaviors in birds, such as aggression and FP (de Haas and van der Eijk, 2018). In the P group, however, the plasma concentration of L-tryptophan was significantly higher than that in the N group by 2.19-log_2−_fold (*p* < 0.05). Moreover, indole and quinolinic acid, downstream metabolites of tryptophan, also had log_2_-fold increases of 1.78 and 2.36, respectively (*p* < 0.05). NE, which acts as a neurotransmitter and as a hormone, is usually activated after exposure to stress and has been found to have increased levels in the blood of HFP birds in response to manual restraint (Korte et al., 1997). In our research, the levels of NE and its precursor tyrosine were higher in the P group than in the N group (*p* < 0.05). Outcomes from various experiments suggest that cognition and memory function are tightly related to fluctuations in the histidine level in the peripheral system (Holeček, 2020). In the P group, the L-histidine concentration was more enriched in plasma than in the N group (*p* < 0.05). Notably, peripheral histidine and aromatic amino acids (AAA, tyrosine, phenylalanine and tryptophan), which had increased levels in P plasma, can be transported into the brain *via* large neutral amino acids (LNAAs) transporter and affect the CNS neurotransmitters histamine, 5-HT, glutamic acid and GABA (Oldendorf et al., 1988). To further investigate the characteristics of the metabolic profile in peckers, we conducted KEGG pathway enrichment analysis of differential metabolites *via* MetabAnaylst. Specifically, 9 metabolic pathways, including ‘glycine, serine and threonine metabolism’, ‘alanine, aspartate and glutamate metabolism’, ‘beta-alanine metabolism’, ‘histidine metabolism’ and ‘tyrosine metabolism’, were identified (Figure 5A; Supplementary Table S2). The peripheric change including glycine, serine and threonine metabolism’, ‘alanine, aspartate and glutamate metabolism’, ‘beta-alanine metabolism’, ‘histidine metabolism’ and ‘tyrosine metabolism’ have previously reported to be associated with depression (Goto et al., 2017; Liu et al., 2020; Johnston et al., 2021; Solís-Ortiz et al., 2021). Next, the association between KEGG metabolic pathways and differential metabolites was exhibited by a network plot (Figure 5B). These enriched metabolic pathways exhibit a complicated interaction involving metabolites, including L-tryptophan, L-histidine and NE.

**Figure 6:**
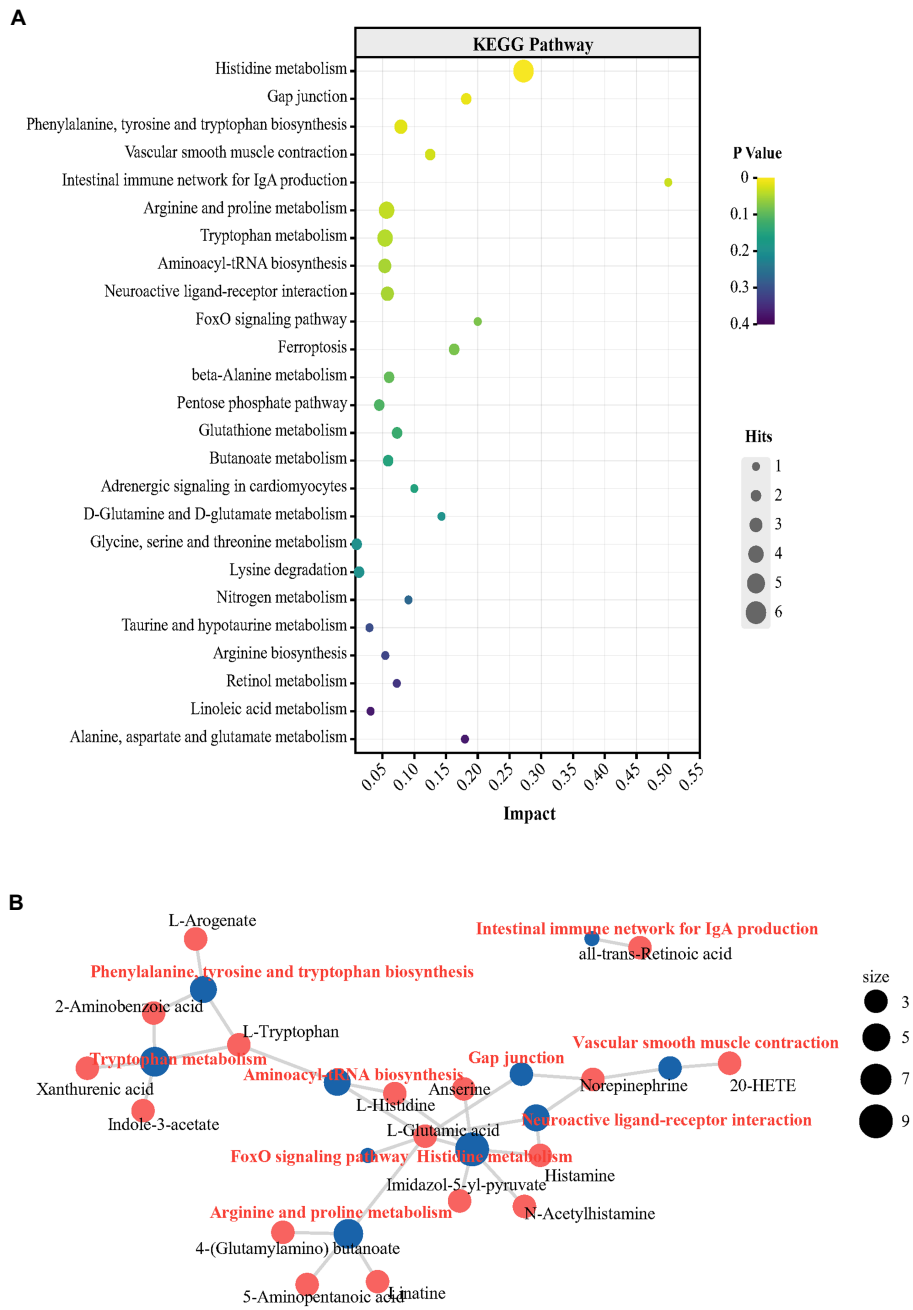
The turbulence of metabolic profile in hippocampus of FP bird. **(A)** Bubble chart from the top 25 KEGG pathways enriching with the differential metabolites in plasma; **(B)** The network plot from the top 10 KEGG pathways enriching with the differential metabolites in hippocampus.

To determine the central effects of the changes in plasma metabolism and the cecal microbial community, hippocampal metabolomics analysis was performed. For subsequent differential metabolite screening and pathway enrichment analysis, multivariate statistical analysis was performed (Supplementary Figures S7–S9). According to the criteria: *p* value < 0.05 and VIP values > 1, 51 differential metabolites were detected in the metabolic profiles of the two groups. In the P group, the levels of 47 metabolites were higher and the levels of 4 metabolites were lower than in the N group (Supplementary Figure S10; Supplementary Table S3). It is worth noting that the L-glutamic acid concentration showed a log_2_-fold increase of 1.51 in the hippocampus in group P (*p* < 0.05). Glutamic acid, which acts as the main excitatory neurotransmitter in the CNS, plays an extensive and key role in the maintenance of brain functions, including emotion and cognition, therefore affecting numerous behaviors, such as aggression, depression and anxiety (Gerhard et al., 2016; Zheng et al., 2019). In the hippocampus of peckers, the concentration of L-tryptophan was also higher than that in the N group (*p* < 0.05). The levels of 5-HT, which is an important product of tryptophan and central neurotransmitters and plays an important role in regulating the onset of FP in laying hens (de Haas and van der Eijk, 2018), did not differ between the two groups. Instead, the levels of xanthurenic acid, one crucial metabolite in the kynurenine (KYN) pathway of tryptophan, significantly increased in the P group (*p* < 0.05). The KYN pathway is an alternate tryptophan breakdown pathway that, under physiological conditions, metabolizes tryptophan (> 95%) into KYN and an array of downstream neuroactive metabolites, including xanthurenic acid (Danielski et al., 2018). Xanthurenic acid, an endogenous kynurenine, is a known vesicular glutamic acid transport (VGLUT) inhibitor and has also been proposed as a mGlu_2/3_ receptor agonist (Neale et al., 2013). Previous studies have found stereoselective blood–brain barrier transport of histidine by *in vivo* or *in vitro* experiments (Nowak et al., 1997; Yamakami et al., 1998). Histamine, which is synthesized from the amino acid histidine through oxidative decarboxylation by histidine decarboxylase in the brain, exerts complicated effects in depression-like and anxiety-like behavior (Raber, 2005). In the present study, the hippocampal levels of L-histidine and histamine were increased in the P group (*p* < 0.05). The decreased NE concentration shown in group P (*p* < 0.05) may have been the result of the competition between peripheral tyrosine (the precursor of NE), other AAAs (phenylalanine and tryptophan) and histidine to LNAA carriers to be transported into the brain (Oldendorf et al., 1988). The KEGG pathway enrichment analysis of differential metabolites showed 9 significantly enriched metabolic pathways, including ‘Histidine metabolism’, ‘Phenylalanine, tyrosine and tryptophan biosynthesis’, ‘Arginine and proline metabolism’, ‘Tryptophan metabolism’ and ‘Aminoacyl-tRNA biosynthesis’ (Figure 6A; Supplementary Table S4). The central alteration, including ‘Histidine metabolism’, ‘Phenylalanine, tyrosine and tryptophan biosynthesis’, and ‘Tryptophan metabolism’ have reported to be associated with depression-like behaviors (Yoshikawa et al., 2014; Wang et al., 2022). The network diagram showed the association between differential metabolites and enrichment pathways (Figure 6B). L-glutamic acid shows a complex relationship with multiple metabolic pathways, suggesting the potentially important role of L-glutamic acid in FP development.

### 3.5. Correlation analysis

**Figure 7:**
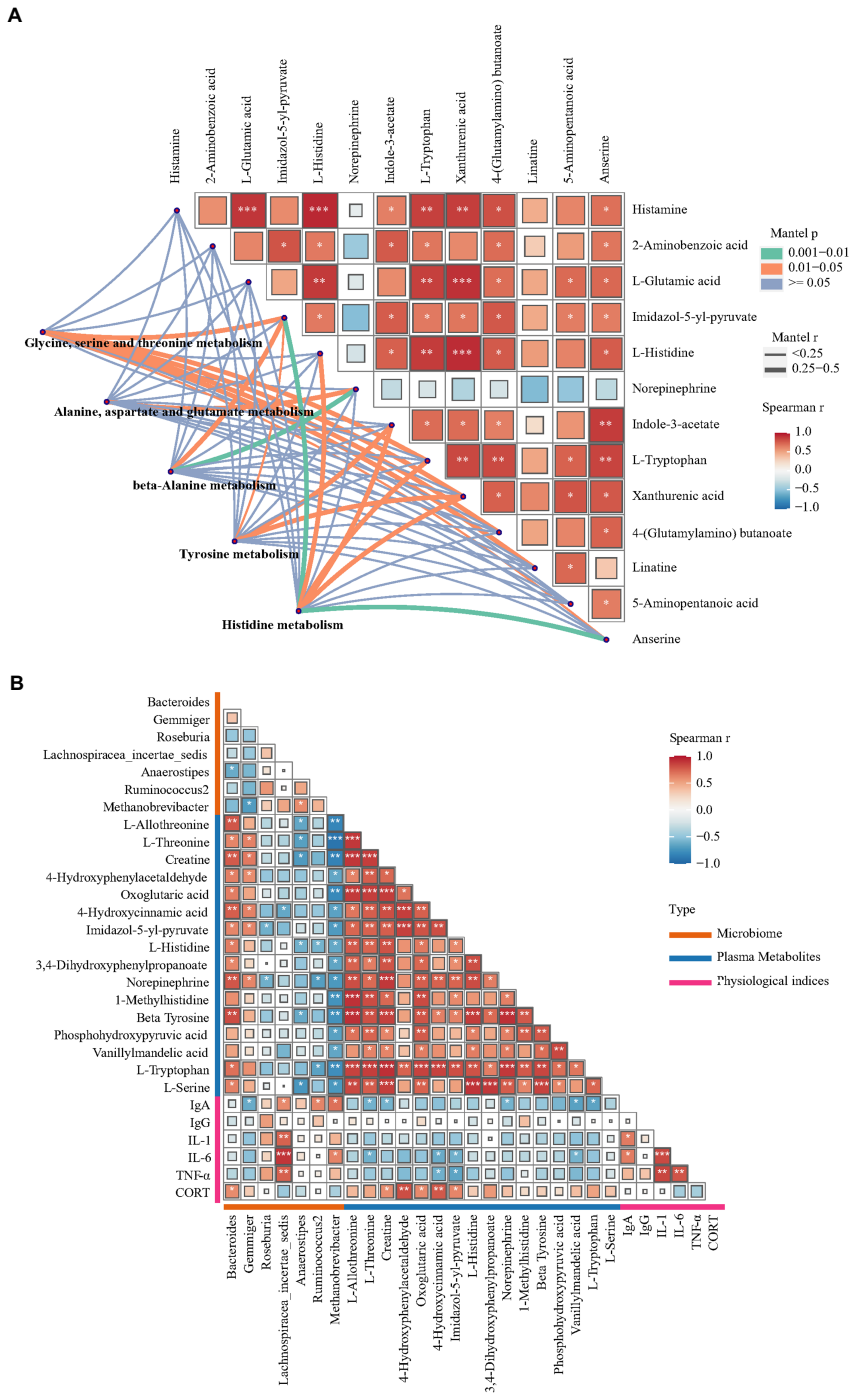
Correlation analysis between gut microorganisms, metabolites and plasma physiological indices. **(A)** Pairwise comparisons of hippocampus metabolites, with a color gradient denoting the Spearman’s correlation coefficients. Plasma KEGG pathways enriched with differential metabolites was correlated with each hippocampus metabolites by partial Mantel tests. Curve width represents the significant correlation coefficients (*p* < 0.05) of the partial Mantel tests. **(B)** Spearman’s correlation analysis between differential microorganism, plasma differential metabolites and plasma physiological indices.

To investigate potential associations among the gut microbiome, plasma physiological index, plasma metabolites and hippocampal metabolites, correlation analysis was conducted. Figure 7A shows the results of Spearman’s correlation analysis performed using the levels of hippocampal differential metabolites and Mantel’s test performed using the levels of hippocampal differential metabolites and plasma metabolic pathways. L-glutamic acid levels showed a significant positive correlation with L-histidine, histamine, L-tryptophan and xanthurenic acid levels in the hippocampus (Spearman *r* > 0.6, *p* < 0.01). A previous study reported an association between glutamic acid and histidine levels (Ritz et al., 2002). The main pathway of histidine catabolism begins with deamination catalyzed by histidase to urocanate and leads through 4-imidazolone-5-propionate and formiminoglutamate to glutamic acid, while the alternative pathways of histidine catabolism include transamination to imidazolepyruvate and decarboxylation to histamine. Increased L-histidine and histamine levels were also found in a rat model of glutamic acid excitotoxicity and other neurodegenerative disorders (Fang et al., 2014). The present study revealed an increase in xanthurenic acid concentration but not in 5-HT levels. Xanthurenic acid is a known VGLUT inhibitor and has also been proposed as a mGlu_2/3_ receptor agonist (Neale et al., 2013). These results indicated that the development of FP is potentially correlated with changes in the glutamatergic system in the CNS. Furthermore, the levels of multiple differential metabolites, including L-histidine, histamine, L-tryptophan and xanthurenic acid, in the hippocampus were significantly correlated with ‘glycine, serine and threonine metabolism’, ‘tyrosine metabolism’ and ‘histidine metabolism’ in plasma. Collectively, these findings indicated that the disturbed glutamatergic system was potentially associated with the differential metabolites involved in ‘glycine, serine and threonine metabolism’ or ‘histidine metabolism’ in plasma. Therefore, a total of 16 differential metabolites, such as L-histidine, L-tryptophan and beta-tyrosine, which participate in ‘glycine, serine and threonine metabolism’, ‘tyrosine metabolism’ and ‘histidine metabolism’, were chosen to perform Spearman correlation analysis with cecal flora genera and plasma physiological indices (Figure 7B). *Bacteroides* and *Methanobrevibacter* abundance showed the closest association with the plasma metabolite profile. L-tryptophan, L-histidine and beta-tyrosine levels were both positively correlated with *Bacteroides* abundance but negatively correlated with *Methanobrevibacter* abundance. *Bacteroides*, the most prominent genus that was enriched in the P group, was associated with abnormal behaviors of the host. The gut microbiome from major depressive disorder patients was found to be enriched with the genus *Bacteroides*, and these microbes were found to be associated with increased anxiety and depression-like behavior and impaired hippocampal neurogenesis in rats subjected to fecal microbiota transplantation (Zhang et al., 2022). Outcomes from numerous experimental methods, including dietary tryptophan restriction and histidine supplementation, have revealed that *Bacteroides* play an important role in histidine metabolism and tryptophan metabolism (Zapata et al., 2018; Kang et al., 2020). More OTUs were enriched in the genus *Methanobrevibacter*, which had decreased abundance in the P group, and its function is closely related to anxiety and depression-like behavior (Chen et al., 2021). Previous research found a negative association between the abundance of *Methanobrevibacter* and dietary tryptophan levels (Rao et al., 2021). Elevated levels of stress hormones, including CORT and N, can also lead to disrupted gut barrier function and altered commensal bacteria (Maes et al., 2013). In this research, the increase in the levels of NE and CORT, which are stress hormones, was positively correlated with *Bacteroides* abundance. However, the potential association between NE, CORT and *Bacteroides* is unclear. To our knowledge, NE levels were found only to change with a significant negative correlation with *Bacteroides* abundance, which was reported in research on depression (Chen et al., 2021).

## 4. Conclusion

Taken together, our results demonstrated the different patterns of the gut microbiota, metabolism and immune system and revealed the potential association between FP, the gut microbiota and the glutamatergic neurotransmitter system. In this research, peckers were found to suffer from long-term stress with a suppressed immune system. Disturbances in the cecal microbiota, including increased *Bacteroides* abundance and decreased *Methanobrevibacter* abundance, were found in peckers. The abundances of the two microorganisms showed significant correlations with the plasma levels of L-tryptophan, beta-tyrosine and L-histidine, which may further affect the hippocampal levels of metabolites involved in the glutamatergic neurotransmitter system, including L-glutamic acid, L-tryptophan, xanthurenic acid, and L-histidine. In conclusion, the findings of this study have provided a new insight into developing novel biotherapeutic strategies for alleviating FP in laying hens.

## Data availability statement

The data presented in the study are deposited in NCBI Sequence Read Archive (SRA) repository (https://www.ncbi.nlm.nih.gov/bioproject/PRJNA933384), accession number PRJNA933384.

## Ethics statement

The animal study was reviewed and approved by the Scientific Ethics Committee of South China Agricultural University.

## Author contributions

CW contributed to the data analysis, investigation, and drafting the manuscript. YL, HW, and ML were responsible for behavioral observation, breeding and sampling. JR provided technical support. XL and YBW were responsible for supervision. YW contributed to the conceptualization, project administration and critical revision of the manuscript. All the authors have read and approved the final manuscript.

## Funding

This work was supported by the National Natural Science Foundation of China (31972610), the Construction Project of Modern Agricultural Science and Technology Innovation Alliance in Guangdong Province (2022KJ128 and 2023KJ128) and the earmarked fund for Modern Agro-industry Technology Research System (CARS-40).

## Conflict of interest

The authors declare that the research was conducted in the absence of any commercial or financial relationships that could be construed as a potential conflict of interest.

## Publisher’s note

All claims expressed in this article are solely those of the authors and do not necessarily represent those of their affiliated organizations, or those of the publisher, the editors and the reviewers. Any product that may be evaluated in this article, or claim that may be made by its manufacturer, is not guaranteed or endorsed by the publisher.

## Footnotes

^1^http://qiime.org/scripts/split_libraries_fastq.html

^2^http://ccb.jhu.edu/software/FLASH/index.shtml

^3^https://www.drive5.com/usearch/manual/uchime_algo.html

^4^https://github.com/torognes/vsearch/releases

^5^https://www.drive5.com/usearch/download.html

^6^http://scikitbio.org/docs/latest/generated/skbio.diversity.alpha.shannon.html#skbio.diversity.alpha.shannon

^7^http://scikit-bio.org/docs/latest/generated/skbio.diversity.alpha.chao1.html#skbio.diversity.alpha.chao1

^8^http://huttenhower.sph.harvard.edu/galaxy/

^9^http://www.hmdb.ca

^10^http://www.massbank.jp/

^11^http://www.lipidmaps.org

^12^https://www.mzcloud.org

^13^http://www.genome.jp/kegg/

^14^https://www.metaboanalyst.ca/
