# Supplementary material for: Differences in peripheral and central metabolites and gut microbiome of laying hens with different feather-pecking phenotypes
Source: Front Microbiol. 2023 Mar 2;14:1132866. doi: 10.3389/fmicb.2023.1132866 (PMC10017472; doi:10.3389/fmicb.2023.1132866)
Supplement: Supplementary file 1 [file Data_Sheet_1.DOCX]

Supplementary Material

1. **Supplementary Figures**


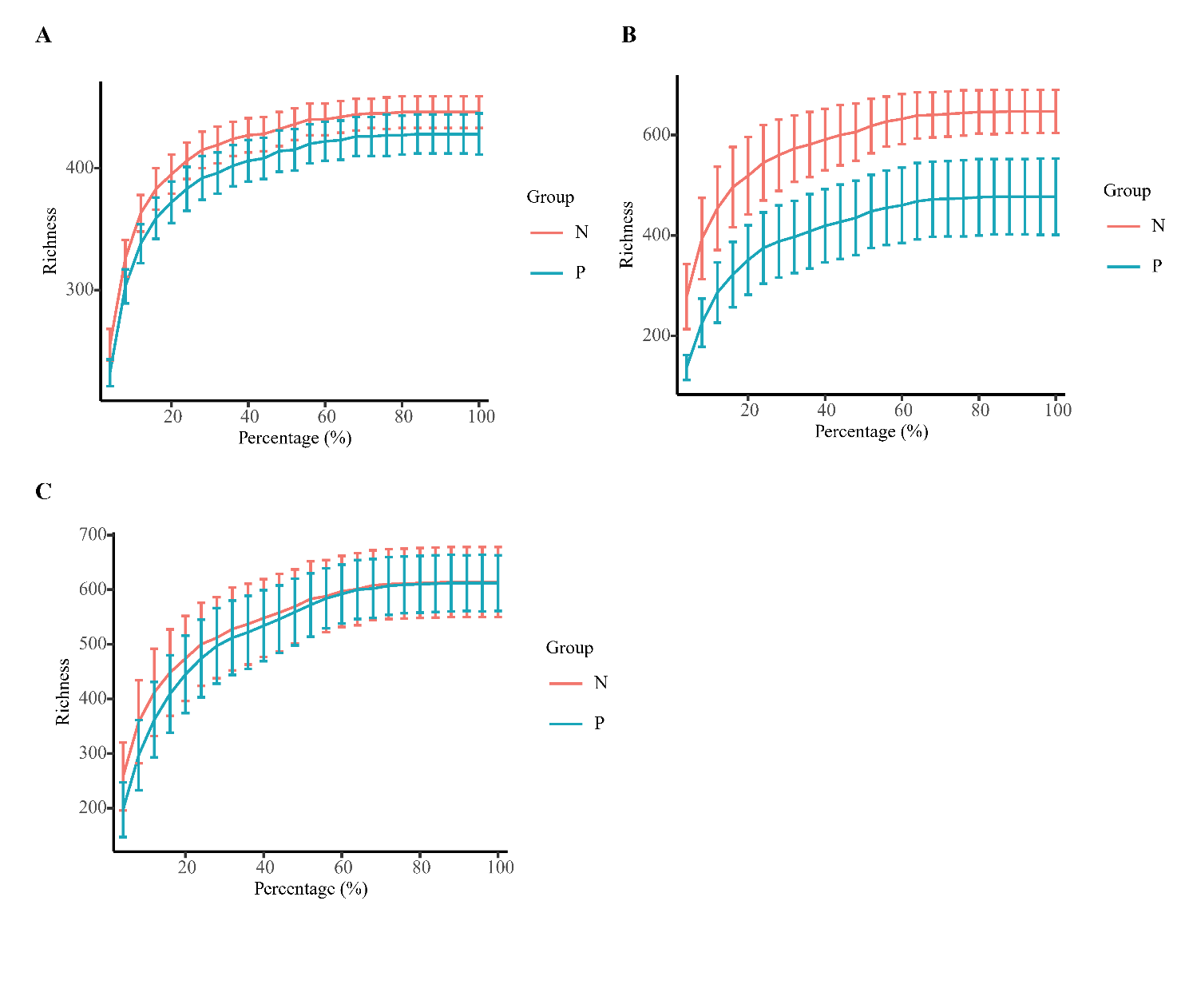


**Supplementary Figure 1.** Rarefaction curve of cecum **(A)**, duodenum **(B)** and ileum **(C)**. P = feather pecker, N = neutral.


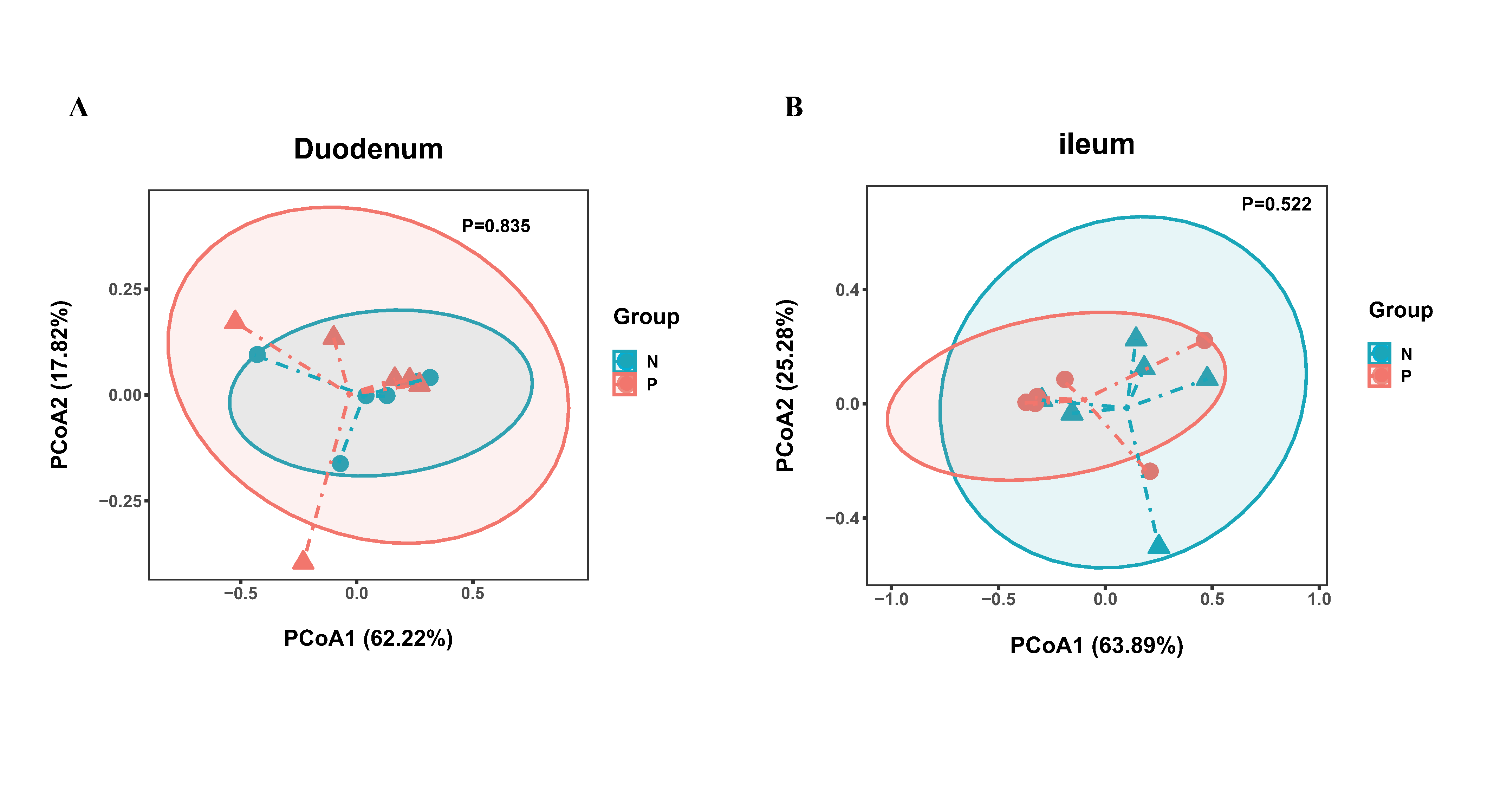


**Supplementary Figure 2.** PCoA analysis of duodenum **(A)** and ileum **(B)** based on the unweighted unifrac distance**.** P = feather pecker, N = neutral.

**
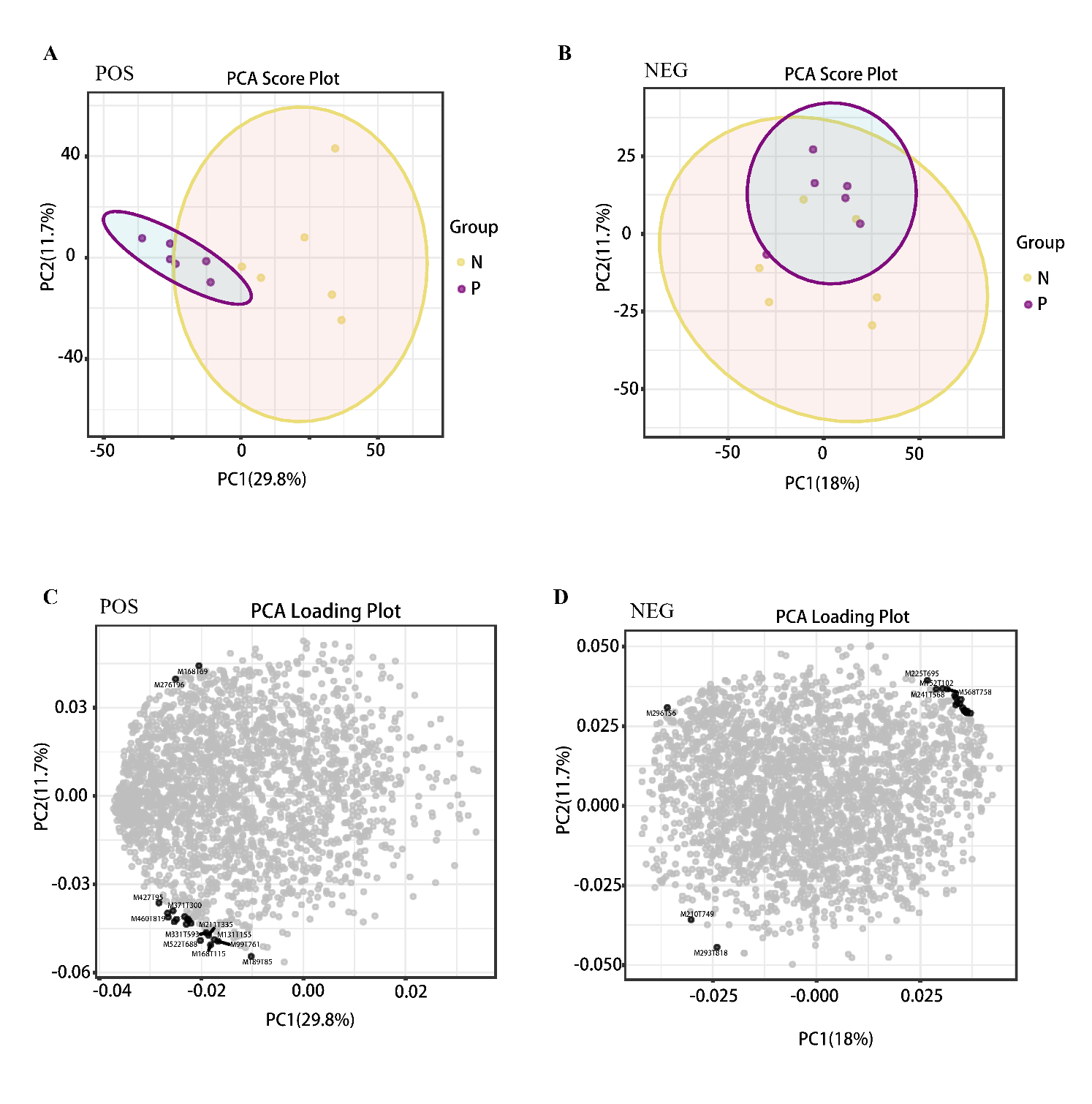
**

**Supplementary Figure 3.** Principal Component Analysis (PCA) of plasma metabolites. **(A,B)** The score plot of metabolic difference between groups P and N by PCA analysis in both positive and negative ion modes. **(C,D)** The loading plot of metabolic difference between groups P and N by PCA analysis in both positive and negative ion modes. P = feather pecker, N = neutral.


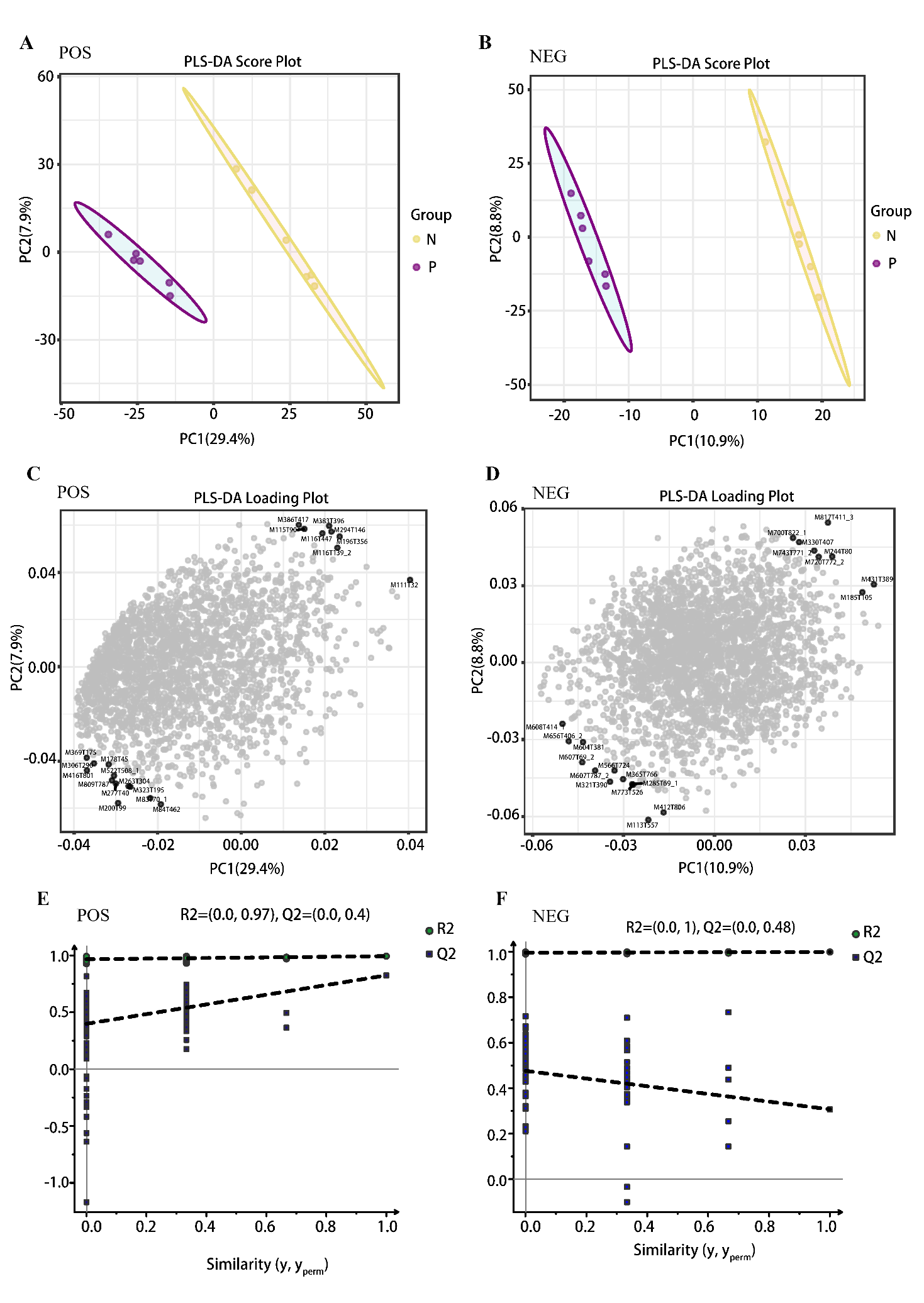
**Supplementary Figure 4.** Partial Least Squares-Discriminate Analysis (PLS-DA) of plasma metabolites. **(A,B)** The score plot of metabolic difference between groups P and N by PLS-DA analysis in both positive and negative ion modes. **(C,D)** The loading plot of metabolic difference between groups P and N by PLS-DA analysis in both positive and negative ion modes. **(E,F)** The permutation test diagram of metabolic difference between groups P and N by PLS-DA analysis in both positive and negative ion modes. P = feather pecker, N = neutral.


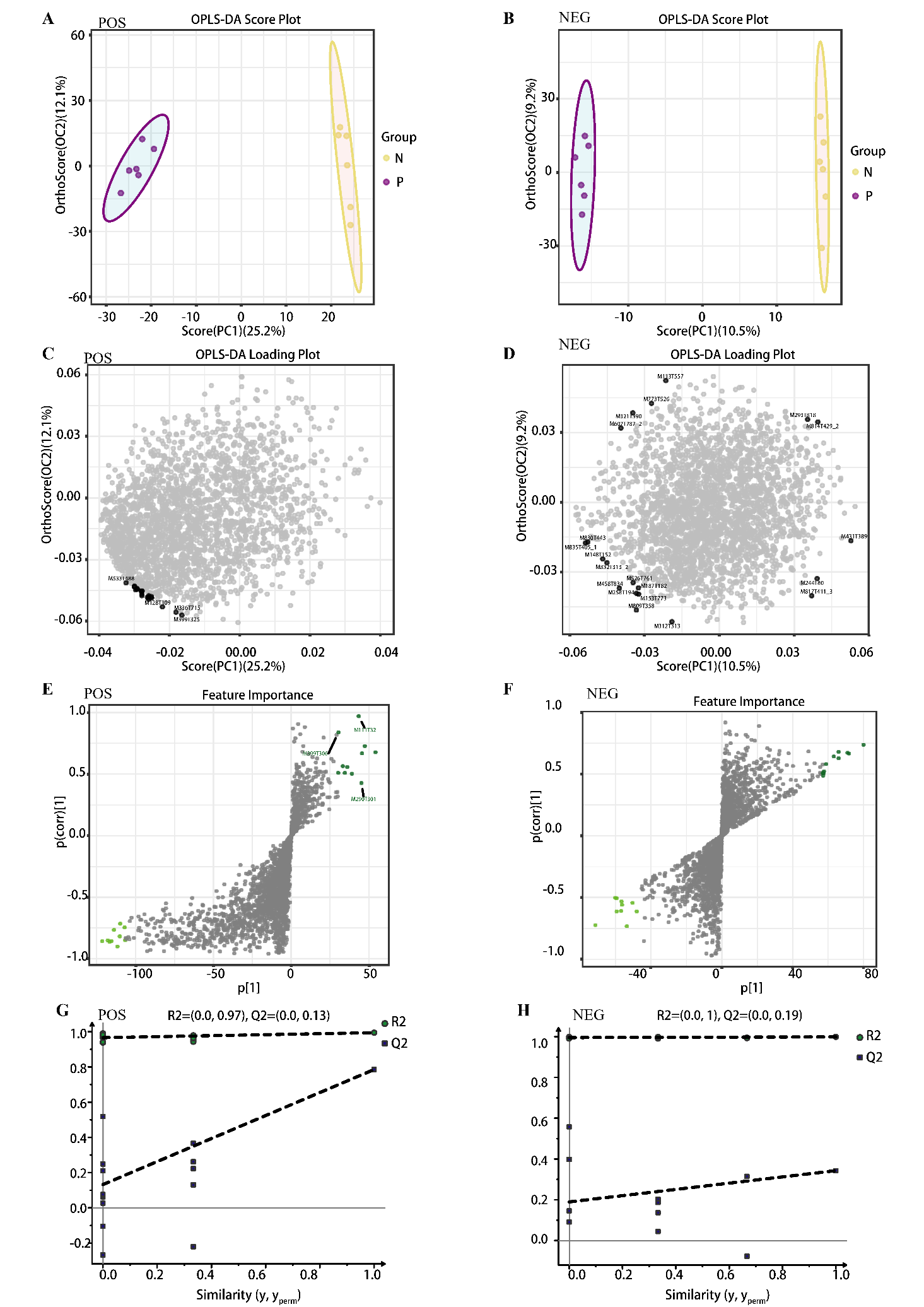


**Supplementary Figure 5.** Orthogonal Projections to Latent Structures Discriminant Analysis (OPLS-DA) of plasma metabolites. **(A,B)** The score plot of metabolic difference between groups P and N by OPLS-DA analysis in both positive and negative ion modes. **(C,D)** The loading plot of metabolic difference between groups P and N by OPLS-DA analysis in both positive and negative ion modes. **(E,F)** the S-plot of metabolic difference between groups P and N by OPLS-DA analysis in both positive and negative ion modes. **(G,H)** The permutation test diagram of metabolic difference between groups P and N by OPLS-DA analysis in both positive and negative ion modes. P = feather pecker, N = neutral.

**
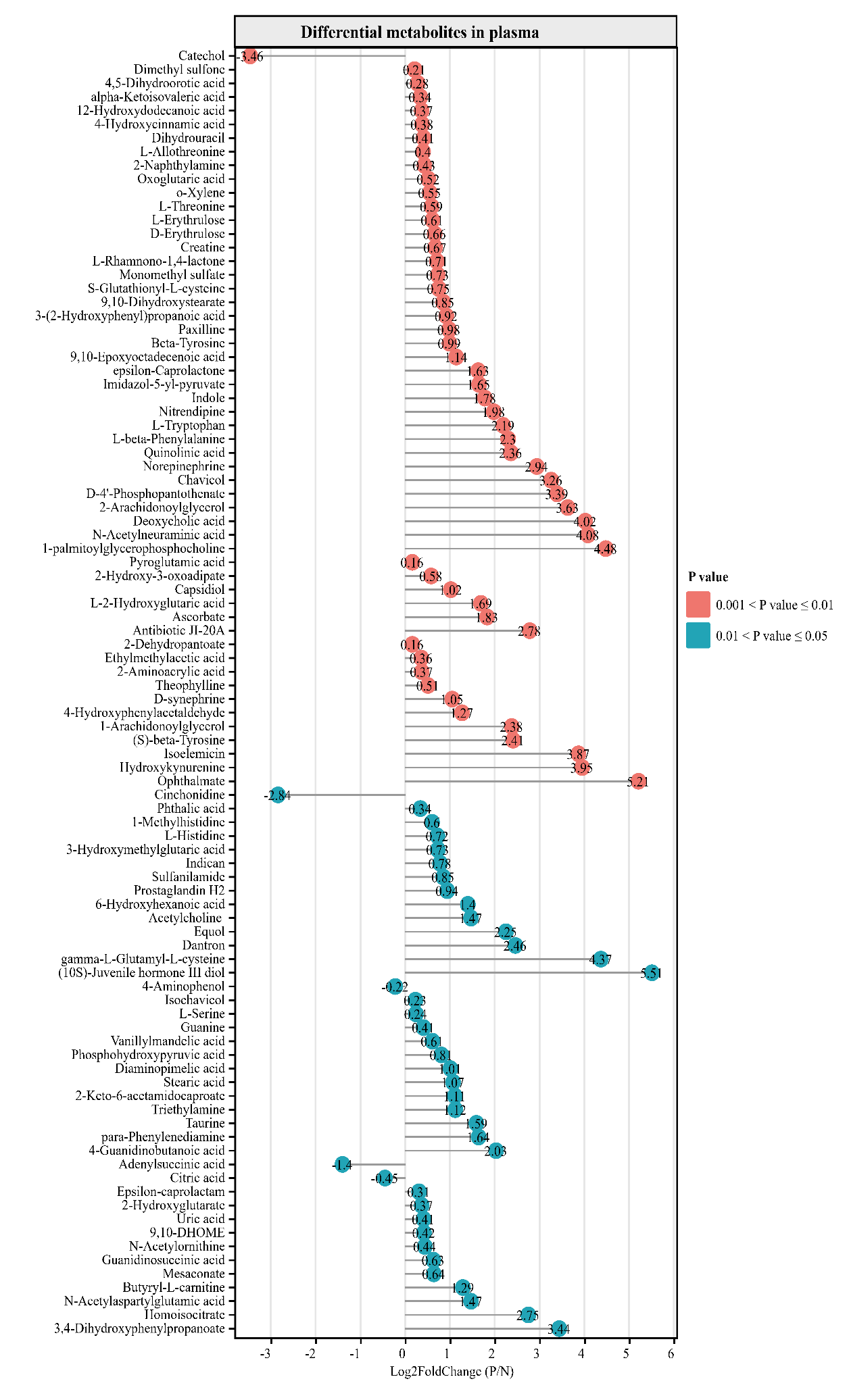
**

**Supplementary Figure 6.** The differential metabolites screened according to the following criteria: P value < 0.05 and VIP values > 1.

**
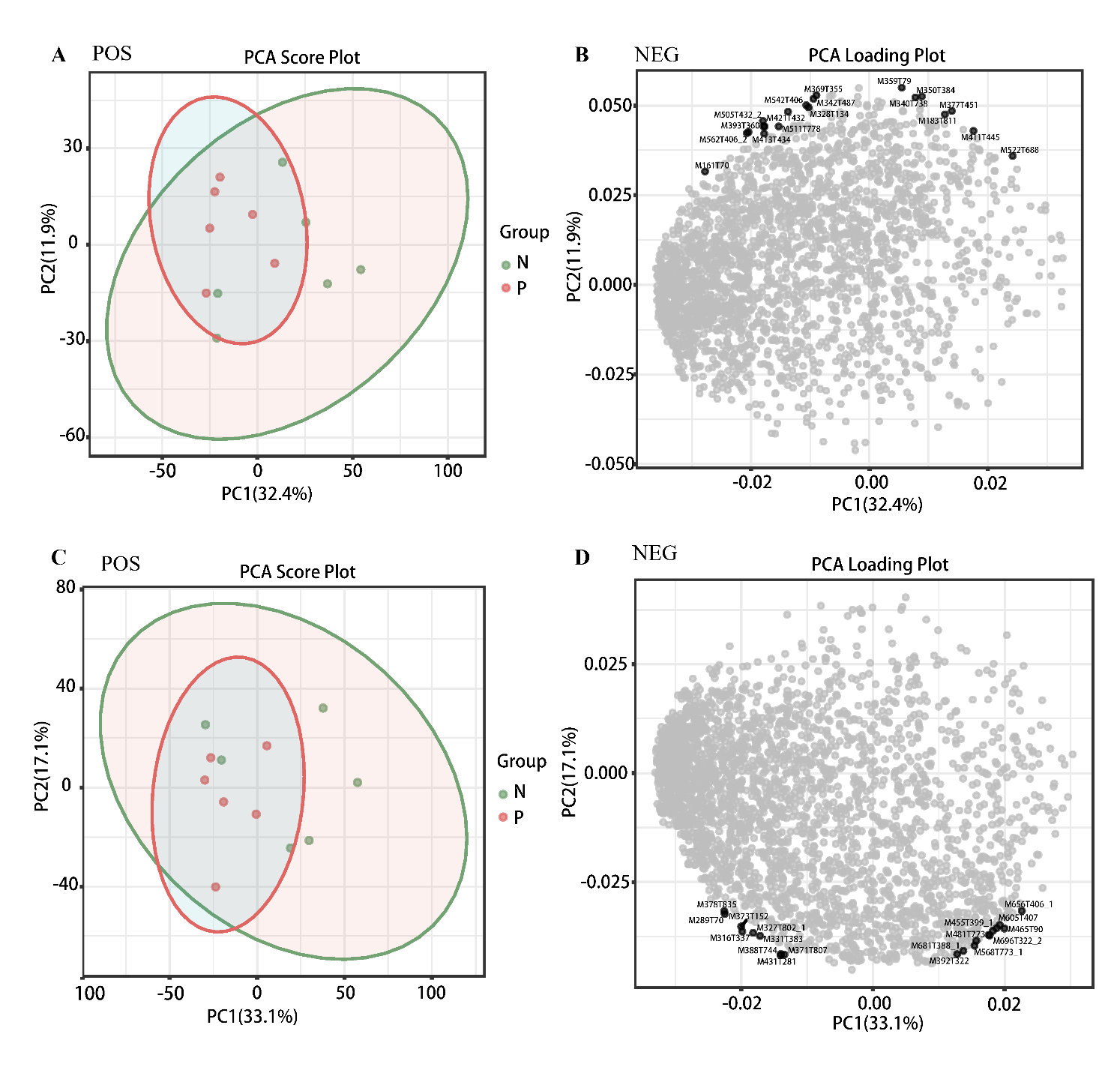
**

**Supplementary Figure 7.** Principal Component Analysis (PCA) of hippocampal metabolites. **(A,B)** The score plot of metabolic difference between groups P and N by PCA analysis in both positive and negative ion modes. **(C,D)** The loading plot of metabolic difference between groups P and N by PCA analysis in both positive and negative ion modes. P = feather pecker, N = neutral.

**
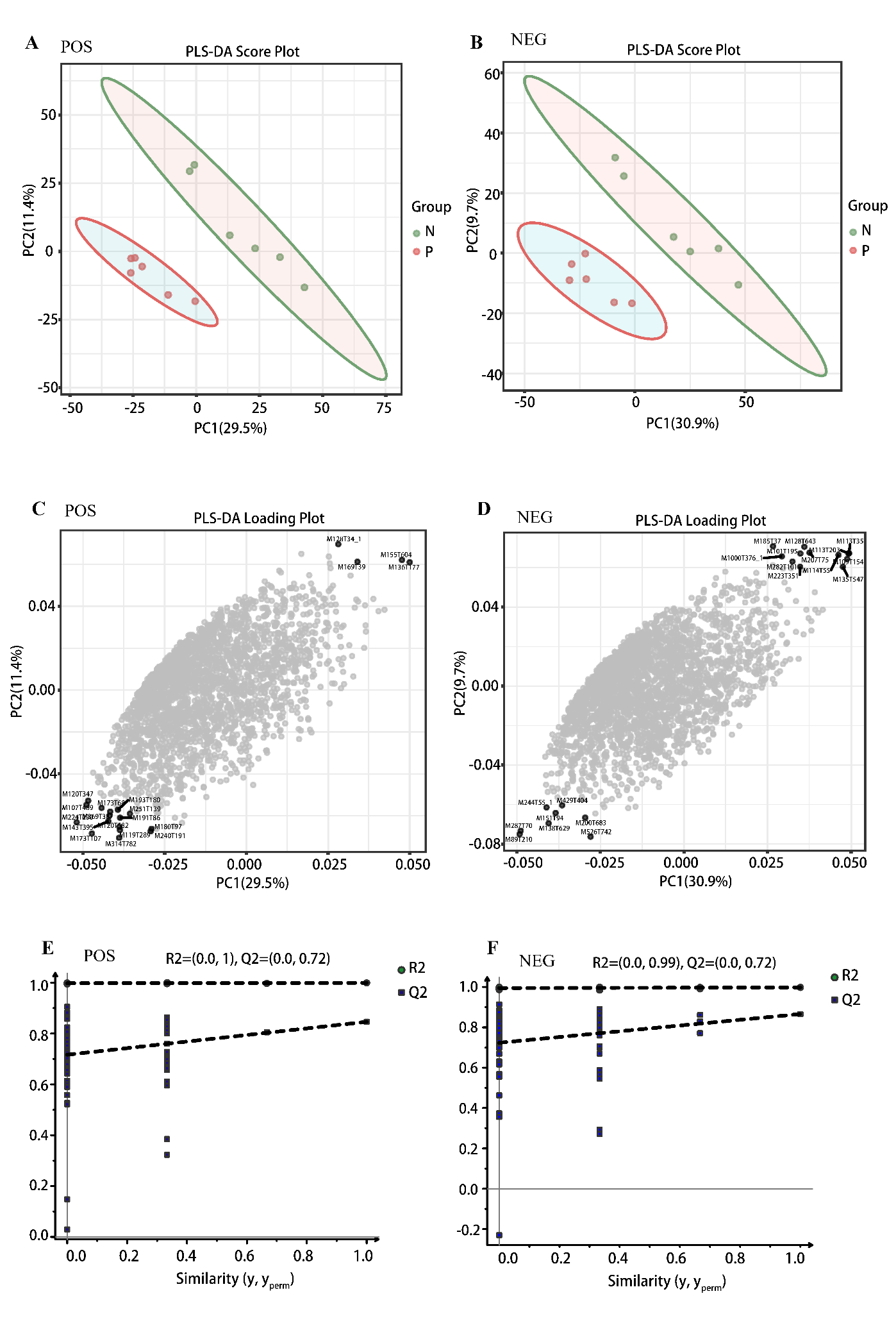
**

**Supplementary Figure 8.** Partial Least Squares-Discriminate Analysis (PLS-DA) of hippocampal metabolites. **(A,B)** The score plot of metabolic difference between groups P and N by PLS-DA analysis in both positive and negative ion modes. **(C,D)** The loading plot of metabolic difference between groups P and N by PLS-DA analysis in both positive and negative ion modes. **(E,F)** The permutation test diagram of metabolic difference between groups P and N by PLS-DA analysis in both positive and negative ion modes. P = feather pecker, N = neutral.

**
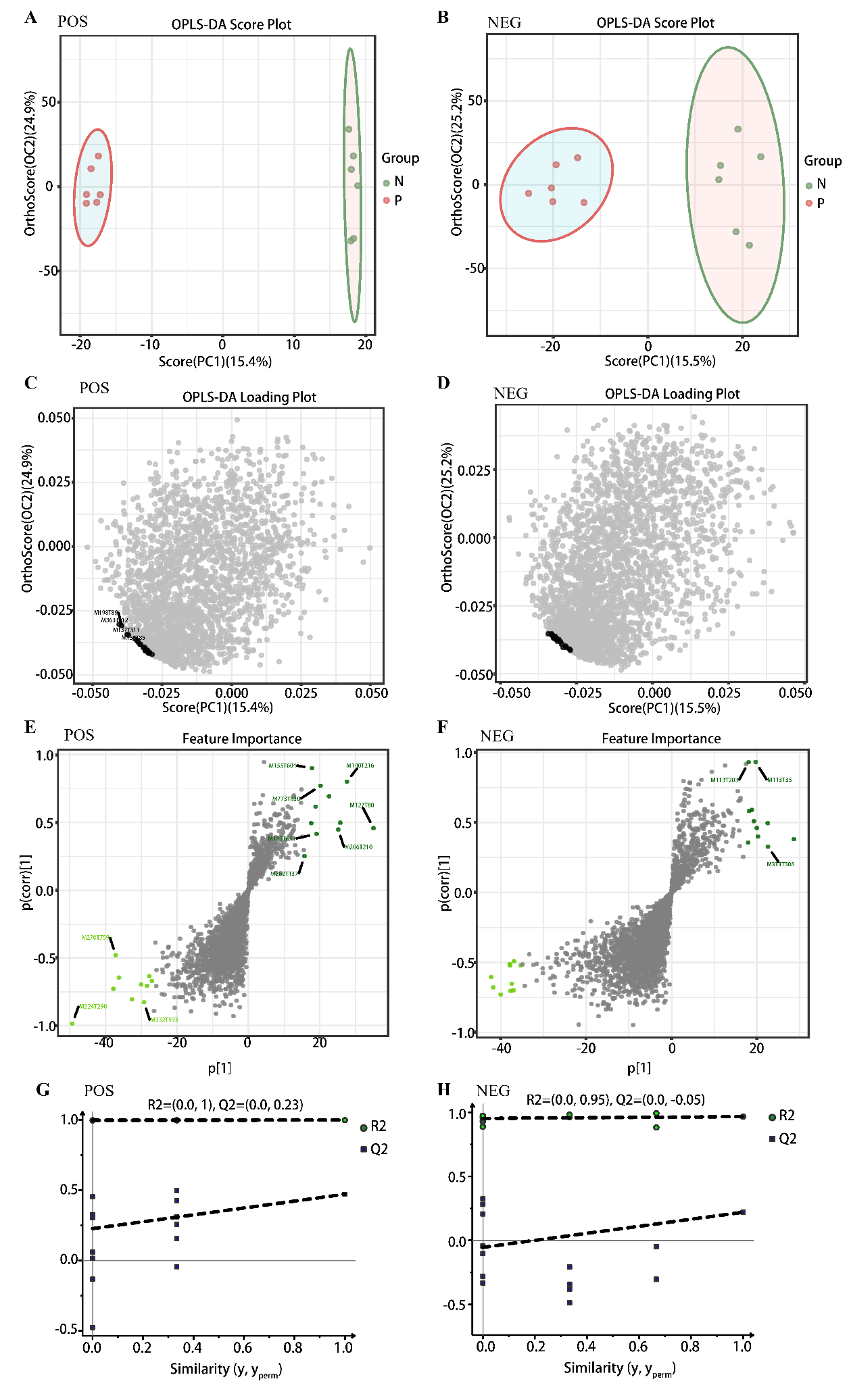
**

**Supplementary Figure 9.** Orthogonal Projections to Latent Structures Discriminant Analysis (OPLS-DA) of hippocampal metabolites. **(A,B)** The score plot of metabolic difference between groups P and N by OPLS-DA analysis in both positive and negative ion modes. **(C,D)** The loading plot of metabolic difference between groups P and N by OPLS-DA analysis in both positive and negative ion modes. **(E,F)** the S-plot of metabolic difference between groups P and N by OPLS-DA analysis in both positive and negative ion modes. **(G,H)** The permutation test diagram of metabolic difference between groups P and N by OPLS-DA analysis in both positive and negative ion modes. P = feather pecker, N = neutral.

**
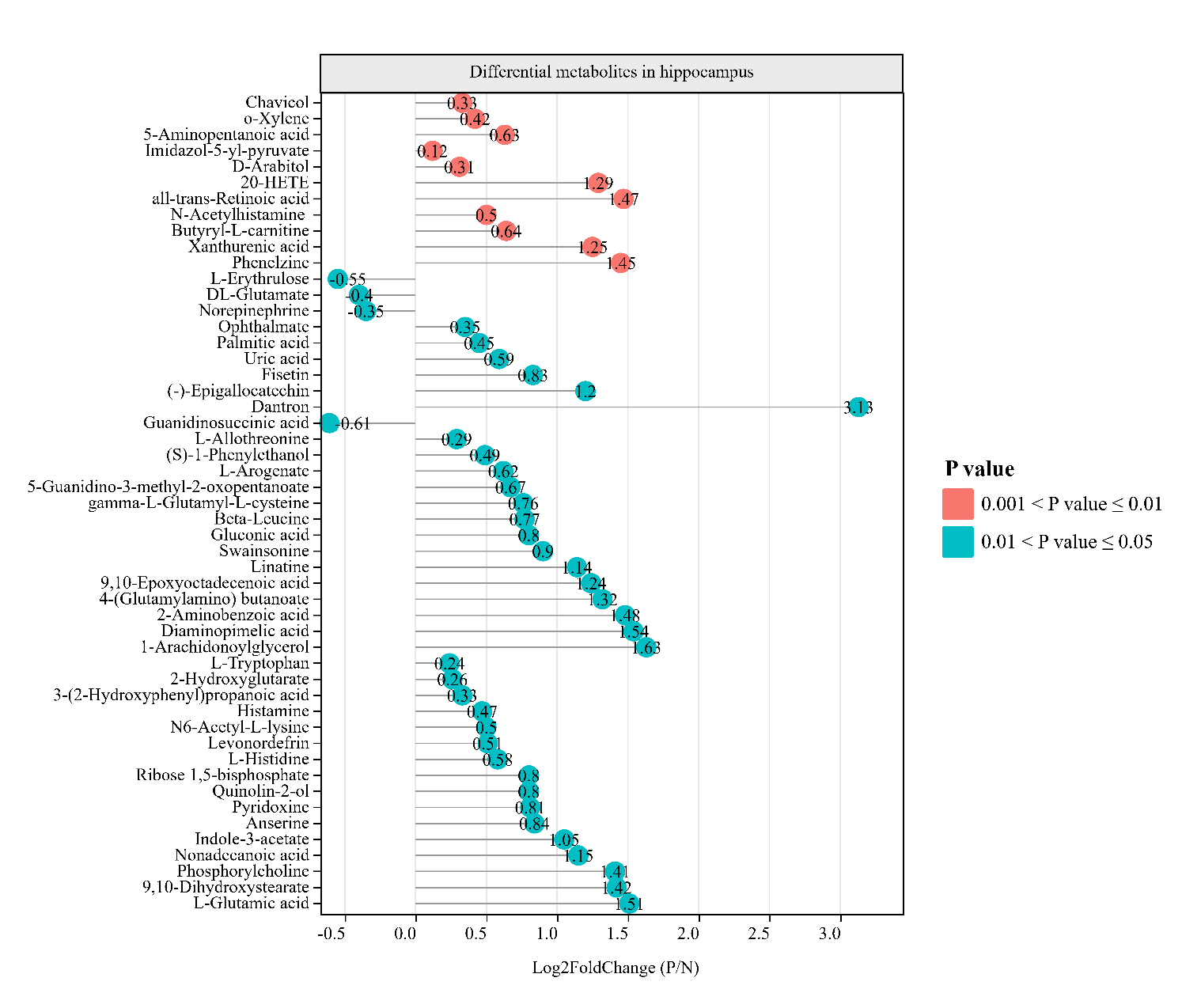
**

**Supplementary Figure 10.** The differential metabolites screened according to the following criteria: P value < 0.05 and VIP values > 1.

1. **Supplementary Tables**

| **Name** | **KEGG** | **log2FC** | **P value** | **VIP** |
| --- | --- | --- | --- | --- |
| (10S)-Juvenile hormone III diol | C16505 | 5.51 | 0.015152 | 1.485843 |
| Ophthalmate | C21016 | 5.21 | 0.008658 | 1.49048 |
| 1-palmitoylglycerophosphocholine | C04102 | 4.48 | 0.002165 | 1.784692 |
| gamma-L-Glutamyl-L-cysteine | C00669 | 4.37 | 0.015152 | 1.303578 |
| N-Acetylneuraminic acid | C19910 | 4.08 | 0.002165 | 1.783666 |
| Deoxycholic acid | C04483 | 4.02 | 0.002165 | 1.742865 |
| Hydroxykynurenine | C02794 | 3.95 | 0.008658 | 1.309082 |
| Isoelemicin | C16975 | 3.87 | 0.008658 | 1.534234 |
| 2-Arachidonoylglycerol | C13856 | 3.63 | 0.002165 | 1.70232 |
| 3,4-Dihydroxyphenylpropanoate | C10447 | 3.44 | 0.041126 | 1.368855 |
| D-4'-Phosphopantothenate | C03492 | 3.39 | 0.002165 | 1.802259 |
| Chavicol | C16930 | 3.26 | 0.002165 | 1.735187 |
| Norepinephrine | C00547 | 2.94 | 0.002165 | 1.692417 |
| Antibiotic JI-20A | C17704 | 2.78 | 0.004329 | 1.661755 |
| Homoisocitrate | C05662 | 2.75 | 0.041126 | 1.139632 |
| Dantron | C10312 | 2.46 | 0.015152 | 1.992079 |
| (S)-beta-Tyrosine | C21308 | 2.41 | 0.008658 | 1.311924 |
| 1-Arachidonoylglycerol | C13857 | 2.38 | 0.008658 | 1.433905 |
| Quinolinic acid | C03722 | 2.36 | 0.002165 | 1.686715 |
| L-beta-Phenylalanine | C20487 | 2.3 | 0.002165 | 1.463584 |
| Equol | C14131 | 2.25 | 0.015152 | 1.536689 |
| L-Tryptophan | C00078 | 2.19 | 0.002165 | 1.431272 |
| 4-Guanidinobutanoic acid | C01035 | 2.03 | 0.025974 | 1.41145 |
| Nitrendipine | C07713 | 1.98 | 0.002165 | 1.767861 |
| Ascorbate | C00072 | 1.83 | 0.004329 | 1.398444 |
| Indole | C00463 | 1.78 | 0.002165 | 1.670477 |
| L-2-Hydroxyglutaric acid | C03196 | 1.69 | 0.004329 | 1.382088 |
| Imidazol-5-yl-pyruvate | C03277 | 1.65 | 0.002165 | 1.387257 |
| para-Phenylenediamine | C19499 | 1.64 | 0.025974 | 1.1767 |
| epsilon-Caprolactone | C01880 | 1.63 | 0.002165 | 1.374838 |
| Taurine | C00245 | 1.59 | 0.025974 | 1.280702 |
| Acetylcholine | C01996 | 1.47 | 0.015152 | 1.190775 |
| N-Acetylaspartylglutamic acid | C12270 | 1.47 | 0.041126 | 1.340225 |
| 6-Hydroxyhexanoic acid | C06103 | 1.4 | 0.015152 | 1.434102 |
| Butyryl-L-carnitine | C02862 | 1.29 | 0.041126 | 1.220451 |
| 4-Hydroxyphenylacetaldehyde | C03765 | 1.27 | 0.008658 | 1.532264 |
| 9,10-Epoxyoctadecenoic acid | C14825 | 1.14 | 0.002165 | 2.256732 |
| Triethylamine | C14691 | 1.12 | 0.025974 | 1.133868 |
| 2-Keto-6-acetamidocaproate | C05548 | 1.11 | 0.025974 | 1.280793 |
| Stearic acid | C01530 | 1.07 | 0.025974 | 1.433832 |
| D-synephrine | C01869 | 1.05 | 0.008658 | 1.568962 |
| Capsidiol | C09627 | 1.02 | 0.004329 | 1.481687 |
| Diaminopimelic acid | C00666 | 1.01 | 0.025974 | 1.248227 |
| Beta-Tyrosine | C04368 | 0.99 | 0.002165 | 1.338252 |
| Paxilline | C13782 | 0.98 | 0.002165 | 2.93631 |
| Prostaglandin H2 | C00427 | 0.94 | 0.015152 | 1.439008 |
| 3-(2-Hydroxyphenyl)propanoic acid | C01198 | 0.92 | 0.002165 | 1.65168 |
| Sulfanilamide | C07458 | 0.85 | 0.015152 | 1.158583 |
| 9,10-Dihydroxystearate | C19622 | 0.85 | 0.002165 | 1.662703 |
| Phosphohydroxypyruvic acid | C03232 | 0.81 | 0.025974 | 1.258243 |
| Indican | C08481 | 0.78 | 0.015152 | 1.362229 |
| S-Glutathionyl-L-cysteine | C05526 | 0.75 | 0.002165 | 1.315994 |
| Monomethyl sulfate | C02704 | 0.73 | 0.002165 | 1.249613 |
| 3-Hydroxymethylglutaric acid | C03761 | 0.73 | 0.015152 | 1.545931 |
| L-Histidine | C00135 | 0.72 | 0.015152 | 1.534177 |
| L-Rhamnono-1,4-lactone | C02991 | 0.71 | 0.002165 | 1.729199 |
| Creatine | C00300 | 0.67 | 0.002165 | 1.833824 |
| D-Erythrulose | C02022 | 0.66 | 0.002165 | 2.986109 |
| Mesaconate | C01732 | 0.64 | 0.041126 | 1.107063 |
| Guanidinosuccinic acid | C03139 | 0.63 | 0.041126 | 1.584669 |
| Vanillylmandelic acid | C05584 | 0.61 | 0.025974 | 1.284009 |
| L-Erythrulose | C02045 | 0.61 | 0.002165 | 2.978794 |
| 1-Methylhistidine | C01152 | 0.6 | 0.015152 | 1.415547 |
| L-Threonine | C00188 | 0.59 | 0.002165 | 1.778824 |
| 2-Hydroxy-3-oxoadipate | C03217 | 0.58 | 0.004329 | 1.681266 |
| o-Xylene | C07212 | 0.55 | 0.002165 | 1.509109 |
| Oxoglutaric acid | C00026 | 0.52 | 0.002165 | 1.771275 |
| Theophylline | C07130 | 0.51 | 0.008658 | 1.571077 |
| 2-Naphthylamine | C02227 | 0.43 | 0.002165 | 1.828513 |
| N-Acetylornithine | C00437 | 0.44 | 0.041126 | 1.093557 |
| 9,10-DHOME | C14828 | 0.42 | 0.041126 | 1.984363 |
| Guanine | C00242 | 0.41 | 0.025974 | 1.31346 |
| Uric acid | C00366 | 0.41 | 0.041126 | 1.261113 |
| L-Allothreonine | C05519 | 0.4 | 0.002165 | 1.883199 |
| Dihydrouracil | C00429 | 0.41 | 0.002165 | 2.926334 |
| 4-Hydroxycinnamic acid | C00811 | 0.38 | 0.002165 | 1.669416 |
| 2-Hydroxyglutarate | C02630 | 0.37 | 0.041126 | 1.390249 |
| 2-Aminoacrylic acid | C02218 | 0.37 | 0.008658 | 1.409034 |
| 12-Hydroxydodecanoic acid | C08317 | 0.37 | 0.002165 | 2.943435 |
| Ethylmethylacetic acid | C18319 | 0.36 | 0.008658 | 1.237886 |
| alpha-Ketoisovaleric acid | C00141 | 0.34 | 0.002165 | 2.865733 |
| Phthalic acid | C01606 | 0.34 | 0.015152 | 1.388119 |
| Epsilon-caprolactam | C06593 | 0.31 | 0.041126 | 1.033808 |
| 4,5-Dihydroorotic acid | C00337 | 0.28 | 0.002165 | 1.740514 |
| L-Serine | C00065 | 0.24 | 0.025974 | 2.080799 |
| Isochavicol | C20464 | 0.23 | 0.025974 | 1.186582 |
| Dimethyl sulfone | C11142 | 0.21 | 0.002165 | 2.507361 |
| Pyroglutamic acid | C01879 | 0.16 | 0.004329 | 2.451338 |
| 2-Dehydropantoate | C00966 | 0.16 | 0.008658 | 1.464456 |
| 4-Aminophenol | C02372 | -0.22 | 0.025974 | 2.058756 |
| Citric acid | C00158 | -0.45 | 0.041126 | 2.043719 |
| Adenylsuccinic acid | C03794 | -1.4 | 0.041126 | 1.751083 |
| Cinchonidine | C11379 | -2.84 | 0.015152 | 1.836921 |
| Catechol | C00090 | -3.46 | 0.002165 | 1.933659 |

**Table 1** The plasm differential metabolites screened according to the following criteria: P value < 0.05 and VIP values > 1.

| **KEGG Pathway Name** | **Total** | **Hits** | **Up hits** | **Down hits** | **P value** |
| --- | --- | --- | --- | --- | --- |
| Glycine, serine and threonine metabolism | 50 | 6 | 6 | 0 | 0.003467384 |
| Alanine, aspartate and glutamate metabolism | 28 | 4 | 2 | 2 | 0.009076237 |
| Pantothenate and CoA biosynthesis | 30 | 4 | 4 | 0 | 0.011604122 |
| beta-Alanine metabolism | 32 | 4 | 4 | 0 | 0.014545845 |
| Neuroactive ligand-receptor interaction | 52 | 5 | 5 | 0 | 0.018936569 |
| Tyrosine metabolism | 78 | 6 | 6 | 0 | 0.028592323 |
| Glyoxylate and dicarboxylate metabolism | 62 | 5 | 4 | 1 | 0.0373076 |
| Cysteine and methionine metabolism | 63 | 5 | 5 | 0 | 0.039589666 |
| Histidine metabolism | 47 | 4 | 4 | 0 | 0.049341152 |
| Aminoacyl-tRNA biosynthesis | 52 | 4 | 4 | 0 | 0.069651587 |
| Sulfur metabolism | 33 | 3 | 3 | 0 | 0.075343517 |
| Glutathione metabolism | 38 | 3 | 3 | 0 | 0.104808139 |
| Citrate cycle (TCA cycle) | 20 | 2 | 1 | 1 | 0.120163671 |
| Taurine and hypotaurine metabolism | 22 | 2 | 2 | 0 | 0.140652226 |
| Regulation of actin cytoskeleton | 5 | 1 | 1 | 0 | 0.141876082 |
| Arginine biosynthesis | 23 | 2 | 2 | 0 | 0.151162896 |
| Valine, leucine and isoleucine biosynthesis | 23 | 2 | 2 | 0 | 0.151162896 |
| Lysine degradation | 50 | 3 | 3 | 0 | 0.189619046 |
| Linoleic acid metabolism | 28 | 2 | 2 | 0 | 0.205634405 |
| Adrenergic signaling in cardiomyocytes | 10 | 1 | 1 | 0 | 0.263807029 |
| Phenylalanine, tyrosine and tryptophan biosynthesis | 34 | 2 | 2 | 0 | 0.273172975 |
| Gap junction | 11 | 1 | 1 | 0 | 0.286051368 |
| D-Glutamine and D-glutamate metabolism | 13 | 1 | 1 | 0 | 0.328564177 |
| Butanoate metabolism | 42 | 2 | 2 | 0 | 0.362730657 |
| Vascular smooth muscle contraction | 16 | 1 | 1 | 0 | 0.387680429 |
| ABC transporters | 138 | 5 | 5 | 0 | 0.40267543 |
| Ascorbate and aldarate metabolism | 50 | 2 | 2 | 0 | 0.448043307 |
| Tryptophan metabolism | 83 | 3 | 3 | 0 | 0.459751821 |
| Caffeine metabolism | 22 | 1 | 1 | 0 | 0.490894874 |
| Sphingolipid metabolism | 25 | 1 | 1 | 0 | 0.535845023 |
| Purine metabolism | 95 | 3 | 2 | 1 | 0.551164783 |
| Pyrimidine metabolism | 65 | 2 | 2 | 0 | 0.589213883 |
| Ferroptosis | 29 | 1 | 1 | 0 | 0.589724503 |
| Arginine and proline metabolism | 78 | 2 | 2 | 0 | 0.688748479 |
| Valine, leucine and isoleucine degradation | 42 | 1 | 1 | 0 | 0.725571639 |
| Primary bile acid biosynthesis | 47 | 1 | 1 | 0 | 0.765005464 |
| Glycerophospholipid metabolism | 52 | 1 | 1 | 0 | 0.798824468 |
| Fructose and mannose metabolism | 54 | 1 | 1 | 0 | 0.810961244 |
| Nicotinate and nicotinamide metabolism | 55 | 1 | 1 | 0 | 0.816755064 |
| Pentose and glucuronate interconversions | 56 | 1 | 1 | 0 | 0.822373141 |
| Fatty acid biosynthesis | 58 | 1 | 1 | 0 | 0.833103012 |
| Phenylalanine metabolism | 60 | 1 | 1 | 0 | 0.843191208 |
| Biosynthesis of unsaturated fatty acids | 74 | 1 | 1 | 0 | 0.898767684 |
| Arachidonic acid metabolism | 75 | 1 | 1 | 0 | 0.901890703 |
| Neomycin, kanamycin and gentamicin biosynthesis | 81 | 1 | 1 | 0 | 0.918724303 |
| Ubiquinone and other terpenoid-quinone biosynthesis | 92 | 1 | 1 | 0 | 0.942501018 |
| Porphyrin and chlorophyll metabolism | 142 | 1 | 1 | 0 | 0.988266881 |

**Table 2** KEGG pathway enrichment analysis of the plasm differential metabolites.

| **Name** | **KEGG** | **log2FC** | **P value** | **VIP** |
| --- | --- | --- | --- | --- |
| Dantron | C10312 | 3.13 | 0.015152 | 1.762669 |
| 1-Arachidonoylglycerol | C13857 | 1.63 | 0.025974 | 1.400077 |
| Diaminopimelic acid | C00666 | 1.54 | 0.025974 | 1.526883 |
| L-Glutamic acid | C00025 | 1.51 | 0.041126 | 1.61098 |
| 2-Aminobenzoic acid | C00108 | 1.48 | 0.025974 | 1.324512 |
| all-trans-Retinoic acid | C00777 | 1.47 | 0.004329 | 2.000984 |
| Phenelzine | C07430 | 1.45 | 0.008658 | 1.821851 |
| 9,10-Dihydroxystearate | C19622 | 1.42 | 0.041126 | 1.806086 |
| Phosphorylcholine | C00588 | 1.41 | 0.041126 | 1.620071 |
| 4-(Glutamylamino) butanoate | C15767 | 1.32 | 0.025974 | 1.721848 |
| 20-HETE | C14748 | 1.29 | 0.004329 | 2.061658 |
| Xanthurenic acid | C02470 | 1.25 | 0.008658 | 1.98795 |
| 9,10-Epoxyoctadecenoic acid | C14825 | 1.24 | 0.025974 | 1.859018 |
| (-)-Epigallocatechin | C12136 | 1.2 | 0.015152 | 1.972555 |
| Nonadecanoic acid | C16535 | 1.15 | 0.041126 | 1.736917 |
| Linatine | C05939 | 1.14 | 0.025974 | 1.811333 |
| Indole-3-acetate | C00954 | 1.05 | 0.041126 | 1.406081 |
| Swainsonine | C10173 | 0.9 | 0.025974 | 1.521523 |
| Anserine | C01262 | 0.84 | 0.041126 | 1.617107 |
| Fisetin | C10041 | 0.83 | 0.015152 | 1.538273 |
| Pyridoxine | C00314 | 0.81 | 0.041126 | 1.461284 |
| Gluconic acid | C00257 | 0.8 | 0.025974 | 1.669085 |
| Quinolin-2-ol | C06338 | 0.8 | 0.041126 | 1.706702 |
| Ribose 1,5-bisphosphate | C01151 | 0.8 | 0.041126 | 1.891488 |
| Beta-Leucine | C02486 | 0.77 | 0.025974 | 1.580435 |
| gamma-L-Glutamyl-L-cysteine | C00669 | 0.76 | 0.025974 | 1.718262 |
| 5-Guanidino-3-methyl-2-oxopentanoate | C20234 | 0.67 | 0.025974 | 1.548369 |
| Butyryl-L-carnitine | C02862 | 0.64 | 0.008658 | 1.967844 |
| 5-Aminopentanoic acid | C00431 | 0.63 | 0.002165 | 1.618543 |
| L-Arogenate | C00826 | 0.62 | 0.025974 | 1.719521 |
| Uric acid | C00366 | 0.59 | 0.015152 | 1.338333 |
| L-Histidine | C00135 | 0.58 | 0.041126 | 1.426149 |
| Levonordefrin | C11768 | 0.51 | 0.041126 | 1.022503 |
| N6-Acetyl-L-lysine | C02727 | 0.5 | 0.041126 | 1.464663 |
| N-Acetylhistamine | C05135 | 0.5 | 0.008658 | 1.612465 |
| (S)-1-Phenylethanol | C11348 | 0.49 | 0.025974 | 1.631312 |
| Histamine | C00388 | 0.47 | 0.041126 | 1.433468 |
| Palmitic acid | C00249 | 0.45 | 0.015152 | 1.762299 |
| o-Xylene | C07212 | 0.42 | 0.002165 | 2.371801 |
| Ophthalmate | C21016 | 0.35 | 0.015152 | 1.798954 |
| Chavicol | C16930 | 0.33 | 0.002165 | 2.078962 |
| 3-(2-Hydroxyphenyl)propanoic acid | C01198 | 0.33 | 0.041126 | 1.578549 |
| D-Arabitol | C01904 | 0.31 | 0.004329 | 2.021253 |
| L-Allothreonine | C05519 | 0.29 | 0.025974 | 1.729136 |
| 2-Hydroxyglutarate | C02630 | 0.26 | 0.041126 | 1.136086 |
| L-Tryptophan | C00078 | 0.24 | 0.041126 | 1.684721 |
| Imidazol-5-yl-pyruvate | C03277 | 0.12 | 0.004329 | 1.975641 |
| Norepinephrine | C00547 | -0.35 | 0.015152 | 1.641157 |
| DL-Glutamate | C00302 | -0.4 | 0.015152 | 1.890539 |
| L-Erythrulose | C02045 | -0.55 | 0.015152 | 1.651302 |
| Guanidinosuccinic acid | C03139 | -0.61 | 0.025974 | 1.959864 |

**Table 3** The hippocampal differential metabolites screened according to the following criteria: P value < 0.05 and VIP values > 1.

| **KEGG Pathway Name** | **Total** | **Hits** | **Up hits** | **Down hits** | **P value** |
| --- | --- | --- | --- | --- | --- |
| Histidine metabolism | 47 | 6 | 6 | 0 | 9.08E-05 |
| Gap junction | 11 | 2 | 1 | 1 | 0.013106 |
| Phenylalanine, tyrosine and tryptophan biosynthesis | 34 | 3 | 3 | 0 | 0.017227 |
| Vascular smooth muscle contraction | 16 | 2 | 1 | 1 | 0.027147 |
| Intestinal immune network for IgA production | 2 | 1 | 1 | 0 | 0.03242 |
| Arginine and proline metabolism | 78 | 4 | 4 | 0 | 0.037154 |
| Tryptophan metabolism | 83 | 4 | 4 | 0 | 0.045113 |
| Aminoacyl-tRNA biosynthesis | 52 | 3 | 3 | 0 | 0.049872 |
| Neuroactive ligand-receptor interaction | 52 | 3 | 2 | 1 | 0.049872 |
| FoxO signaling pathway | 5 | 1 | 1 | 0 | 0.079127 |
| Ferroptosis | 29 | 2 | 2 | 0 | 0.080346 |
| beta-Alanine metabolism | 32 | 2 | 2 | 0 | 0.095201 |
| Pentose phosphate pathway | 35 | 2 | 2 | 0 | 0.110777 |
| Glutathione metabolism | 38 | 2 | 2 | 0 | 0.126972 |
| Butanoate metabolism | 42 | 2 | 2 | 0 | 0.14937 |
| Adrenergic signaling in cardiomyocytes | 10 | 1 | 0 | 1 | 0.152105 |
| D-Glutamine and D-glutamate metabolism | 13 | 1 | 1 | 0 | 0.193138 |
| Glycine, serine and threonine metabolism | 50 | 2 | 2 | 0 | 0.196195 |
| Lysine degradation | 50 | 2 | 2 | 0 | 0.196195 |
| Nitrogen metabolism | 19 | 1 | 1 | 0 | 0.269449 |
| Taurine and hypotaurine metabolism | 22 | 1 | 1 | 0 | 0.304904 |
| Arginine biosynthesis | 23 | 1 | 1 | 0 | 0.316343 |
| Retinol metabolism | 25 | 1 | 1 | 0 | 0.33867 |
| Linoleic acid metabolism | 28 | 1 | 1 | 0 | 0.370827 |
| Alanine, aspartate and glutamate metabolism | 28 | 1 | 1 | 0 | 0.370827 |
| Vitamin B6 metabolism | 29 | 1 | 1 | 0 | 0.381201 |
| Fatty acid elongation | 40 | 1 | 1 | 0 | 0.484812 |
| Fatty acid degradation | 50 | 1 | 1 | 0 | 0.564119 |
| Glycerophospholipid metabolism | 52 | 1 | 1 | 0 | 0.578478 |
| Pentose and glucuronate interconversions | 56 | 1 | 1 | 0 | 0.60582 |
| Phosphonate and phosphinate metabolism | 56 | 1 | 1 | 0 | 0.60582 |
| Fatty acid biosynthesis | 58 | 1 | 1 | 0 | 0.618831 |
| Phenylalanine metabolism | 60 | 1 | 1 | 0 | 0.63142 |
| Glyoxylate and dicarboxylate metabolism | 62 | 1 | 1 | 0 | 0.643602 |
| Cysteine and methionine metabolism | 63 | 1 | 1 | 0 | 0.649544 |
| ABC transporters | 138 | 2 | 2 | 0 | 0.667753 |
| Biosynthesis of unsaturated fatty acids | 74 | 1 | 1 | 0 | 0.708823 |
| Arachidonic acid metabolism | 75 | 1 | 1 | 0 | 0.713697 |
| Tyrosine metabolism | 78 | 1 | 0 | 1 | 0.727843 |
| Neomycin, kanamycin and gentamicin biosynthesis | 81 | 1 | 1 | 0 | 0.741303 |
| Purine metabolism | 95 | 1 | 1 | 0 | 0.795967 |
| Porphyrin and chlorophyll metabolism | 142 | 1 | 1 | 0 | 0.908786 |

**Table 4** KEGG pathway enrichment analysis of the hippocampal differential metabolites.
